# Modeling Indoor Inorganic Aerosol Concentrations During the ATHLETIC Campaign with IMAGES

**DOI:** 10.1021/acsestair.4c00060

**Published:** 2024-09-03

**Authors:** Bryan Berman, Bryan Cummings, Hongyu Guo, Pedro Campuzano-Jost, Jose Jimenez, Demetrios Pagonis, Douglas Day, Zachary Finewax, Anne Handschy, Benjamin A. Nault, Peter DeCarlo, Shannon Capps, Michael Waring

**Affiliations:** † Department of Civil, Architectural and Environmental Engineering, Drexel University, Philadelphia, Pennsylvania 19104, United States; ‡ Department of Chemistry and Cooperative Institute for Research in Environmental Sciences (CIRES), University of Colorado, Boulder, Boulder, Colorado 80309, United States; § Department of Chemistry and Biochemistry, Weber State University, Ogden, Utah 84408, United States; ∥ Center for Aerosol and Cloud Chemistry, Aerodyne Research, Inc., Billerica, Massachusetts 01821, United States; ⊥ Department of Environmental Health and Engineering, Johns Hopkins University, Baltimore, Maryland 21218, United States

**Keywords:** Inorganic aerosols, Indoor modeling, ATHLETIC, IMAGES, ISORROPIA

## Abstract

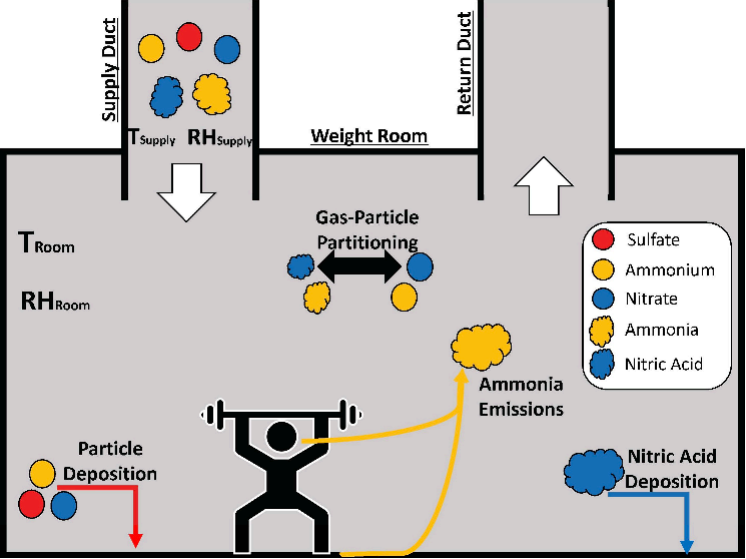

In 2018, the ATHLETIC campaign was conducted at the University of Colorado Dal Ward Athletic Center and characterized dynamic indoor air composition in a gym environment. Among other parameters, inorganic particle and gas-phase species were alternatingly measured in the gym’s supply duct and weight room. The Indoor Model of Aerosols, Gases, Emissions, and Surfaces (IMAGES) uses the inorganic aerosol thermodynamic equilibrium model, ISORROPIA, to estimate the partitioning of inorganic aerosols and corresponding gases. In this study herein, measurements from the ATHLETIC campaign were used to evaluate IMAGES’ performance. Ammonia emission rates, nitric acid deposition, and particle deposition velocities were related to observed occupancy, which informed these rates in IMAGES runs. Initially, modeled indoor inorganic aerosol concentrations were not in good agreement with measurements. A parametric investigation revealed that lowering the temperature or raising the relative humidity used in the ISORROPIA model drove the semivolatile species more toward the particle phase, substantially improving modeled-measured agreement. One speculated reason for these solutions is that aerosol water was enhanced by increasing the RH or decreasing the temperature. Another is that thermodynamic equilibrium was not established in this indoor setting or that the thermodynamic parametrizations in ISORROPIA are less accurate for typical indoor settings. This result suggests that applying ISORROPIA indoors requires further careful experimental validation.

## Abstract 2

This work applies an indoor aerosol model, IMAGES, that estimates the partitioning of inorganic aerosol components and their corresponding gas-phase species with ISORROPIA by leveraging measurements from a university athletic center and derived relationships between occupancy and nitric acid deposition, particle deposition, and ammonia emissions. This study highlights that applying ISORROPIA indoors can sometimes result in inaccurate gas-particle partitioning. However, forcing the model to predict increased particle water by either adjusting relative humidity up or temperature down will result in accurate gas-particle partitioning.

## 1 Introduction

Residents of industrialized countries spend most of their time indoors where they are exposed to air pollution from indoor sources or of outdoor origin.^1^ One major class of these pollutants includes particulate matter (PM), which is causally associated with morbidity and mortality^2−5^ and is composed of organic aerosols (OA) and inorganic aerosols (IA).^6−8^ Major components of IA are sulfate (), ammonium (), and nitrate (), which interact with inorganic gases such as ammonia (NH_3_) and nitric acid (HNO_3_). Due to differences in source and loss processes between indoor and outdoor environments, some pollutants may exist at much higher or lower concentrations indoors than in the ambient air.^2^

NH_3_ is one example of a pollutant that often exists at higher concentrations indoors than outdoors due to substantial indoor sources.^9−12^ Indoor NH_3_ is often sourced from certain cooking and cleaning activities, emissions from building materials, and emissions from occupants.^9,13−18^ Indoor NH_3_ emissions are essential to understand since NH_3_ contributes to the formation of IA and because NH_3_ influences gas-to-particle partitioning by neutralizing acidic species.^19−21^ Recent decreases in the use of NH_3_-based cleaning products and increased use of low-emitting building materials may cause building occupants to be the dominant source of indoor NH_3_.^19^ Thus, the effects of human-emitted NH_3_ on indoor air quality have become a topic of interest.^22^

Beko et al.^23^ outlined the Indoor Chemical Human Emissions and Reactivity (ICHEAR) project, which examined the role of human emissions on indoor chemistry. As part of this project, Li et al.^19^ characterized how human NH_3_ emission rates varied in a test chamber as a function of temperature (*T*), relative humidity (RH), human subject age and clothing characteristics, and ozone (O_3_) concentration. They found that NH_3_ emissions were affected mainly by *T*, age of the human subject, and clothing, but these emissions rates were negligibly influenced by RH and O_3_. As part of the ATHLETic center study of Indoor Chemistry (ATHLETIC) field campaign, Finewax et al.^24^ investigated the impacts of human exercise on NH_3_ emissions as well as other activities on different species. They concluded that an occupant’s NH_3_ emissions increased with their metabolic rate.

Previously, Berman et al.^25^ incorporated the IA thermodynamic equilibrium model, ISORROPIA,^26,27^ into the existing Indoor Model of Aerosols, Gases, Emissions, and Surfaces (IMAGES)^28,29^ framework to better consider indoor IA partitioning and concentrations. ISORROPIA simulates the gas-particle partitioning of inorganic species with known values of temperature, RH, and total concentrations (gas + particle) of inorganic species and is described in Section 2.2. IMAGES is a platform that initially only simulated organic aerosol (OA) concentration, composition, partitioning behavior, and secondary formation using the 2D-volatility basis set framework, which replicates thermodynamic principles provided by OA absorptive partitioning theory.^30−32^ Berman et al.^25^ extended IMAGES to incorporate the inorganic aerosol thermodynamic equilibrium model, ISORROPIA. Berman et al.^25^ tested the model against measured data in a classroom^33^ and used *T* and the difference between indoor and outdoor CO_2_ (ΔCO_2_, ppm to estimate NH_3_ concentrations from occupants. However, rigorous evaluation of the approach was difficult since the validation measurements did not include concentrations of NH_3_ or HNO_3_, which also precluded evaluating whether ISORROPIA predicted the IA partitioning well.^33^

The work herein builds upon Berman et al.^25^ by first evaluating ISORROPIA partitioning with measured indoor concentrations. Results will show that ISORROPIA required either a decrease in the input *T* or an increase in the input RH for simulated partitioning to agree with measurements, since either change pushed aerosol species concentrations toward the particle phase. Next, particle, gas, and occupancy data from the ATHLETIC campaign were used to derive relationships of net NH_3_ emissions, the deposition velocity of particles, and the deposition velocity of HNO_3_ to observed dynamic occupancy. Using these relationships and the ATHLETIC campaign’s robust measurements of inorganic species, occupancy, and environmental conditions, the application of IMAGES with the adjusted thermodynamic inputs was evaluated.

## 2 Methods

### 2.1 ATHLETIC Campaign Measurements

Measurements from the ATHLETIC campaign defined the scope of this work. Using various instruments described by Claflin et al.^34^ and in Table 1 of Finewax et al.,^24^ time-resolved measurements of *T*, RH, , , , NH_3_, HNO_3_, and CO_2_ were taken in the Dal Ward Athletic Center’s weight room and supply duct University of Colorado, Boulder. Specifically, an Aerodyne HR-TOF-AMS measured nonrefractory particle composition (i.e., , ,  less than 1 μm in aerodynamic diameter),^35^ an Aerodyne/TOFWERK I-CIMS measured HNO_3_, a Picarro SI2108 measured NH_3_, and a Picarro G2401 measured CO_2_, supply temperature and RH.^24,34,36−39^ These instruments were placed on the balcony and a nearby supply air register and alternated between the two sampling locations every 5–10 min. Detailed information about the measurement campaign and the instrumentation used are described in detail in Finewax et al.^24^ and Claflin et al.^34^ However, the limits of detection can be found in Table S1. The aerosol richinorganic species might contain some organic contribution,^40,41^ although that effect is likely small for this data set based on the analysis in this work (Section 3.3).^42^ The University of Colorado (CU) Facilities management provided room temperature, and room RH was derived by Claflin et al.^34^ by using building temperature, local pressure, and H_2_O mixing ratio measured by the Picarro instruments. The weight room’s temperature was controlled at ∼293 K; this value was assumed when any weight room temperature data was missing.

The weight room’s volume was estimated to be 1700 m^3^ with a constant supply airflow of 200  delivered by the building’s heating, ventilation, and air conditioning (HVAC) system, resulting in a room air exchange rate of ∼7 h ^–1^. Occupants throughout time were counted from video recordings of the gym’s main room and balcony.^24^ This modeling study considered the portion of the campaign from November 7–19, 2018, corresponding to the timesteps where complete concentration data in the supply duct and weight room (i.e., concentration measurements of , , , NH_3_, and HNO_3_ in the supply duct and weight room), and humidity data in the weight room were all available. However, HNO_3_ measurements were missing in the supply duct for 12 h on November 10, and supply duct NH_3_ concentrations were missing between November 12 and November 15. Since these values are needed for the IMAGES modeling, the portion of the data set where these significant data gaps existed were not included in the IMAGES simulations. The preprocessing necessary to provide a uniform time step for use in IMAGES as well as the time periods of the ATHLETIC campaign modeled with IMAGES are provided in Section S1 of Supporting Information (SI). Additionally, how often inorganic concentration measurements go below the limits of detection are provided in Table S2.

ATHLETIC campaign measurements were used to evaluate ISORROPIA’s performance in indoor environments (Section 2.2), explore drivers of modeled-measured agreement for ISORROPIA in our data set (Section 2.3), and selectively constrain IMAGES (Section 2.4), which also necessitated deriving deposition and emission rates as a function of occupancy (Section 2.5). Given the species measured, particulate matter is assumed to be entirely composed of ammonium-sulfate, ammonium-nitrate, and water.

### 2.2 ISORROPIA Evaluation Using Weight Room Measurements

ISORROPIA is an inorganic aerosol thermodynamic equilibrium model that estimates the gas-to-particle partitioning of IA species when given *T*, RH, and total (gas + particle) concentrations and is described in detail elsewhere.^26,27^ However, to briefly summarize, ISORROPIA formulates the aerosol-gas partitioning problem formulated as either forward or reverse. In forward problems, known values of temperature, RH, and total (gas + aerosol) concentrations of sodium, sulfate, ammonium, nitrate, chloride, magnesium, potassium, and calcium are used to calculate the gas phase concentrations of ammonia (NH_3_), hydrochloric acid (HCl), and nitric acid (HNO_3_), as well as the aerosol concentrations of hydrogen (H^+^), sodium (Na^+^), , bisulfate (), , , CL^–^, calcium (Ca^2+^), potassium (K^+^), magnesium (Mg^2+^), and water (H_2_O).^26^ When solving the reverse problem, ISORROPIA uses the aerosol phase concentrations of the inputs to calculate the corresponding gas phase concentrations of species in equilibrium.^26,27^ This work uses the forward mode since measurements of , , , NH_3_, and HNO_3_ are given (Section 2.1), thus fully constraining the sulfate-nitrate-ammonium system. Additionally, the aerosol can be in a thermodynamically metastable or stable state. In the metastable case, salts do not precipitate under supersaturated conditions. Therefore, aerosols will always be aqueous.^26,27^ In the latter, salts precipitate if saturation is exceeded; thus, aerosols can exist as solid or aqueous species.^26,27^

Since ISORROPIA is used in atmospheric models such as GEOS-Chem and the Community Multiscale Air Quality model (CMAQ), it has been evaluated extensively with outdoor measurements.^26,27,43−49^ However, Berman et al.^25^ represents the only known indoor application of ISORROPIA to explicitly simulate indoor thermodynamics; yet, that work excluded a comprehensive evaluation of the applicability of ISORROPIA indoors because NH_3_ and HNO_3_ measurements were not available for that study. Since these gases were measured by the ATHLETIC campaign, the applicability of ISORROPIA to this indoor environment was comprehensively evaluated here.

To do so, ISORROPIA was used to partition the total (i.e., gas + particle concentrations, where gas and particle concentrations were measured separately) concentration of each inorganic species in the room air between the aerosol and gas phase whenever complete data was available (∼96% of the time during November 7–19, 2018). The resulting modeled aerosol and gas phase concentrations were then compared to room air measurements. ISORROPIA’s metastable mode, which prevents saltation (solid formation) in the aerosol phase, was used during this evaluation. Since saltation is kinetically limited, using ISORROPIA’s metastable mode is a reasonable assumption for this fast-changing environment. Still, an evaluation using ISORROPIA’s stable mode, which allows particles to be aqueous or solids and did not produce markedly better agreement, is shown in Section S2 of the SI.

### 2.3 Optimizing Indoor Environmental Conditions To Be Used in IMAGES

Because we observed during this phase that the ISORROPIA-partitioned  and  concentrations were often underpredicted using the provided room *T* and RH (RH_room,meas_) directly (Section 3.1), a parametric study of the influences of *T* and RH on chemical partitioning was done with ISORROPIA. Both lower *T* and higher RH increase the tendency for species to condense. Thus, modifying *T* or RH will shift the partitioning in ISORROPIA. ISORROPIA was executed with each unique combination of *T* and RH over the entire timeseries of measured room concentrations. Results from this parametric test are discussed in Section S2 and were used to inform the environmental conditions necessary to produce satisfactory agreement between the model and measurements.

ISORROPIA compared better with the measured indoor concentrations at specific *T* and RH combinations within the parametric test described in the previous paragraph. Since ISORROPIA simulates the IA partitioning in IMAGES, the *T* and RH values fed to ISORROPIA were chosen to minimize the partitioning error as defined by the difference of the modeled partitioning fractions from the measured (though the reasons for this error were indiscoverable in this study design) rather than reflecting actual conditions. The indoor *T* was reasonably constant (∼293 K) during the ATHLETIC campaign (Section 2.1), so the indoor RH was optimized for a constant *T* of 293 K to best match the chemical partitioning.

The RH value that resulted in the best partitioning agreement between the volatile species (RH_room,op__t_) was determined by first calculating the weighted averages (ε_room_) of measured and ISORROPIA-modeled indoor particle fraction of  () and  () (i.e, ). The ε_room_ was calculated to combine all the partitioning information in a single metric for each measurement time:
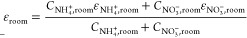
1where  and () are the concentrations of  and  in the weight room. Next, using an orthogonal regression, statistics for the line of best fit (i.e., the correlation coefficient, *R*^2^, the slope, *m*, and the *y*-intercept, *b*) between measured and ISORROPIA-modeled ε_room_ were calculated and used to compute the distance (*d*) between a perfect one-to-one correlation (where *R*^2^ = 1, *m* = 1, and *b* = 0), and the actual correlation:
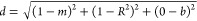
2Finally, the case where *d* was at a minimum was chosen as the optimal condition, which at 293 K was an RH of 98%. Although an RH of 98% is not a realistic indoor value, this value was determined algorithmically to provide the best partitioning agreement. Therefore, setting the RH to 98% in this work makes up for a missing kinetic term, whose source is unclear. Therefore, results using this value are shown in Section 3.1. More details concerning the results of this optimization technique can be found in Section S3.

### 2.4 IMAGES Model Overview

**Figure 1:**
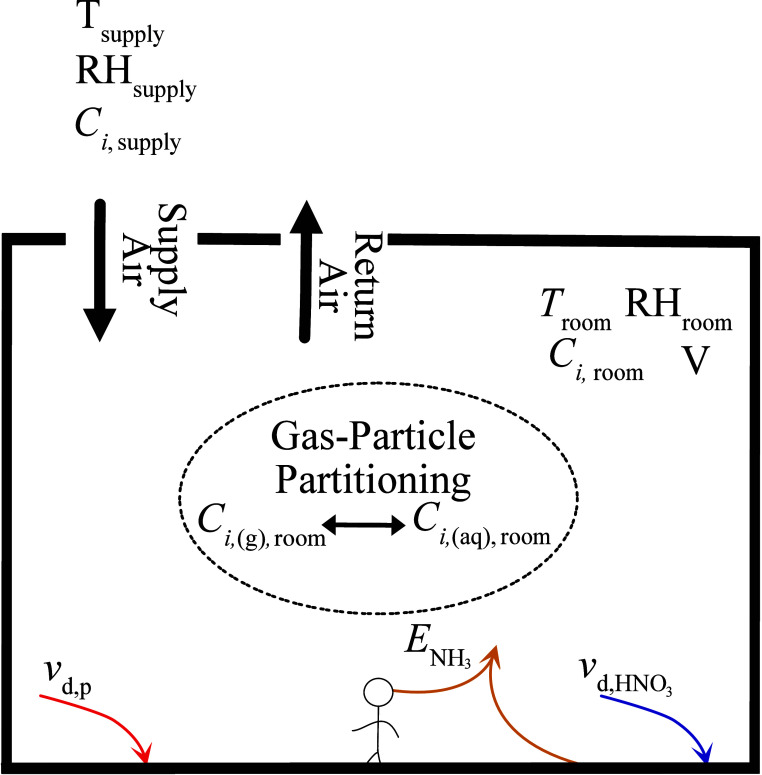
Schematic of IMAGES as applied to modeling the ATHLETIC Campaign. Temperature, RH, and inorganic particle and gas concentrations were measured in the supply duct and room. Modeled processes in the space include particle deposition, HNO_3_ deposition, and NH_3_ emissions. ISORROPIA determined gas-particle partitioning.

The comprehensive indoor thermodynamic particle model, IMAGES, was employed to simulate IA concentrations in the weight room. Specifically, IMAGES uses a well-mixed box model to inform a mass balance that describes total (including gas and particle phases) indoor concentrations of any contaminant species *i* (*C*_*i*,room_, ) when given its source rate (*S*_*i*_, ) and first-order loss rate coefficient (*l*_*i*_, h^–1^):
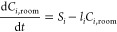
3IMAGES uses ISORROPIA to estimate gas- and particle-phase fractions for each IA species.^25^ Specifically, the concentrations of , , , NH_3_, and HNO_3_ were modeled to simulate the measurements from the ATHLETIC campaign. A schematic that illustrates how IMAGES and ISORROPIA interact is displayed in Figure 1.

Measurements were taken in the supply duct and weight room of a gym (Section 2.1), and so the modeled source and loss rates reflect those observations. The weight room was designed to have a constant volume flow delivered by the building’s HVAC system. When air is supplied to a room, gases and particles from the supply duct are introduced as a source. Additional indoor sources of NH_3_ exist, including emissions from building materials or occupants (which are elevated during exercise).^24,34^ Therefore, the source rate, *S*_*i*_, for each total quantity (gas + particle) of species, *i*, is
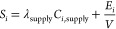
4where  is the supply air exchange rate;  is the supply duct concentration; *V* (m^3^) is the room volume; and  ( is the net indoor emission rate.

Air is assumed to leave the weight room through an HVAC return at the same rate as it is supplied. Particles and gases either are removed with the return air or deposit onto surfaces.^50−53^

Therefore, the loss rate, *l*_*i*_ (h^–1^) of each total quantity (gas + particle) of species, *i*, is defined as
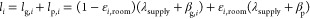
5where *l*_g,*i*_ represents gas-phase losses; *l*_p,*i*_ represents particle-phase losses; β_p_ (h^–1^) and *β*_g,*i*_ (h^–1^) are the net particle and gas deposition rates; and ε_*i*,room_ is the particle fraction of species *i* in the weight room, as determined by ISORROPIA.^54,55^

### 2.5 Relating Emissions and Deposition Rates to Occupancy

Emission and deposition parameters directly impact indoor gas and particle concentrations and may vary with occupancy. Thus, estimates of *E*_NH3_, the net deposition velocity of particles (), and the net deposition velocity of HNO_3_ () were derived as functions of the number of occupants or the change in indoor CO_2_ concentrations from estimated outdoor concentrations, ΔCO_2_ (Section S3). Specifically, the emission and deposition rates were computed at every time step by constraining eqs 3–5 with measured values of the supply duct and room concentrations. A surface-area-to-volume ratio  of 2.5 m^–1^ was assumed to obtain  and  ( from β_p_ and β_HNO3_ (h^–1^), respectively, which was informed by Manuja et al.^24^ In this procedure, β_p_ was computed first by considering only measured  because it is nonvolatile and, thus, has no gas-phase sources or losses,^2,33^ leaving β_p_ as the only unknown variable in the mass balance. Assuming internally mixed particles (i.e., β_p_ applies uniformly to all species),^56^ this time-resolved β_p_ could then be used as input to determine  at each measurement time since  is the only unknown variable in the mass balance on  and HNO_3_. Similarly, using the mass balance on  and NH_3_, the net emission rate, , can be determined.

It was hypothesized that the inferred , , and  would increase as a function of occupancy.^9,54^ Linear regressions were used to develop functional forms of each parameter based on occupancy values. Only data points where all concentrations fell above the detection limit were considered when creating these relationships. If a concentration value fell below the detection limit, that data points and the one at the previous time step, which informs the deposition and emission values (Section S4), were removed from the linear regression. As such, 2.2% of the data was removed from the linear regressions with this method. The limit of detection values for each species can be found in Table S1 of the SI. Relationships for when no values were removed from the linear regression are also included in Section S4. These linear relationships were used to provide  and  as a function of occupancy or ΔCO_2_ when running IMAGES. However, since the correlation between *v*_d,p_ and both occupancy and ΔCO_2_ was so low, a constant value taken from the average *v*_d,p_ (0.0054 ) was used instead.

## 3 Results and Discussion

### 3.1 Indoor ISORROPIA Evaluation

ISORROPIA was first run in a standalone evaluation using the measured inorganic species and environmental conditions to directly evaluate ISORROPIA’s ability to recreate the observed indoor IA partitioning. Since measured room concentrations were used directly, neither IMAGES nor its mass balance parameters were utilized for this evaluation.

**Figure 2:**
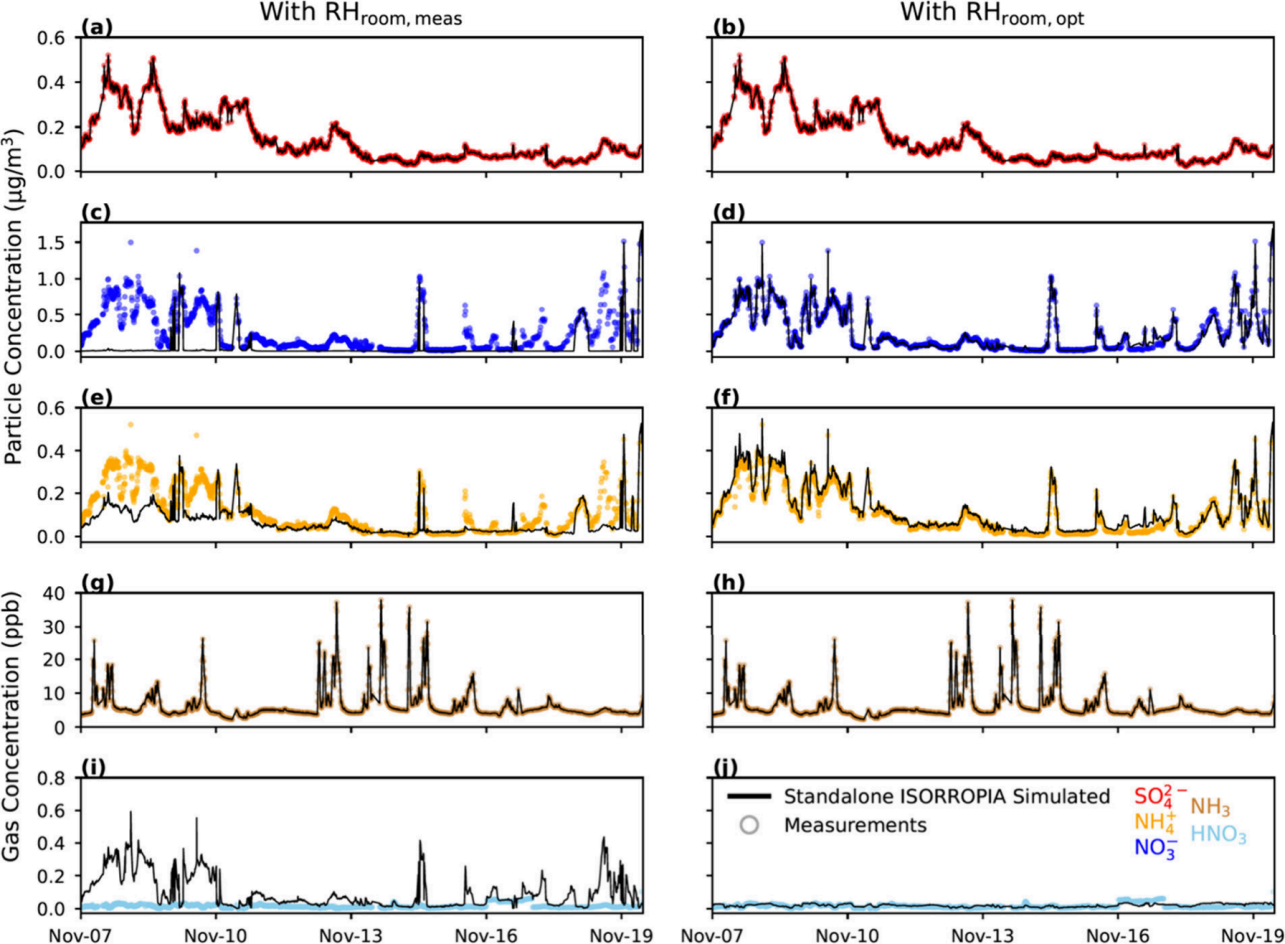
Time series of standalone ISORROPIA simulated (black line) particle and gas concentrations using RH_room,meas_ (left column) and RH_room,opt_ (right column). Measured concentrations (circle markers) are shown for comparison.

**Figure 3:**
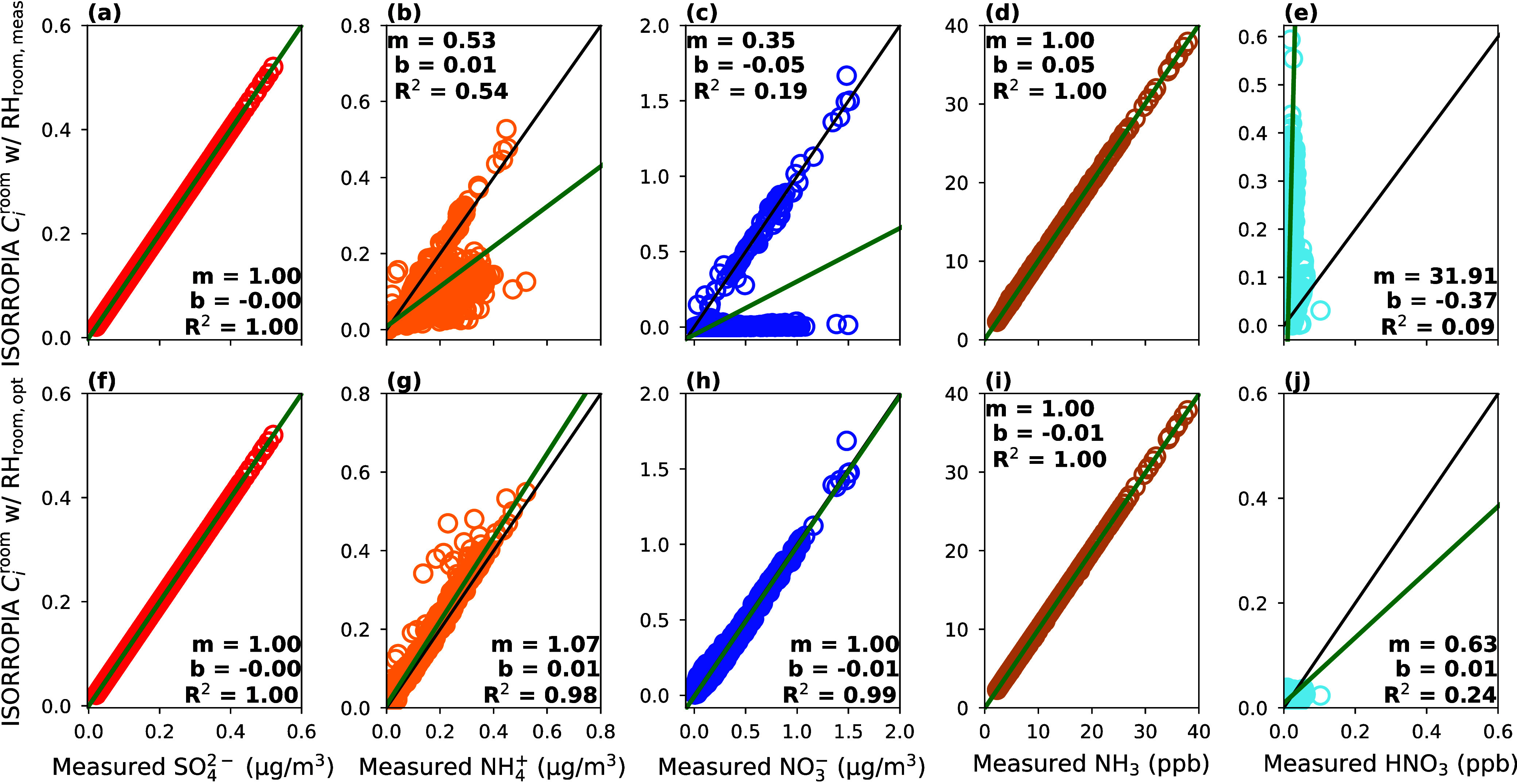
Comparison of standalone ISORROPIA simulated concentrations against measured concentrations using RH_room,meas_ (a–e) and RH_room,opt_ (f–j) as inputs to ISORROPIA. The green line represents the line of best fit calculated with an orthogonal regression, while the black line is the 1:1 line. The correlation coefficient, *R*^2^; slope, *m*; and *y*-intercept, *b*, are displayed for each regression.

Figures 2 and 3 include the evaluation of ISORROPIA against measured concentrations with either measured or optimized *T* and RH values. For these runs, the  consistently agreed strongly between measured and ISORROPIA-partitioned fractions (Figures 2a and 3a) since  is nonvolatile and, thus, always in the particle phase.^2^ However, ISORROPIA did not estimate , HNO_3_, and  concentrations in good accordance with observations when the real-time RH_room,meas_ was used as input. When RH_room,meas_ was used as input to ISORROPIA, it frequently predicted that nitrate would be gaseous when particle-bound nitrate was observed in reality (Figure S3). For instance, Figure 2c,I shows that the ISORROPIA-partitioned HNO_3_ values closely align with the measured  at various times between Nov 7 and Nov 10. This inaccurate partitioning leads to a considerable overprediction of HNO_3_ and underprediction of  (Figure 3c,e). Still, HNO_3_ may not be a meaningful metric to compare given its low concentration magnitude.

Similarly, when RH_room,meas_ was used as input to ISORROPIA,  was underpredicted often (Figure 2e). Specifically,  was always underpredicted when  was simulated to be completely evaporated. Since  was measured at low concentrations, an underprediction of it by ISORROPIA resulted in a large relative error (Figures 2e and 3b). Nevertheless, measured and ISORROPIA-partitioned NH_3_ were in good agreement (Figure 3d), which is possible given the excess NH_3_ attributable to indoor sources.

The ISORROPIA evaluation improves significantly when using RH_room,opt_ of 98% (Figures 2 (right column) and 3f–j). According to the best-fit statistics, HNO_3_ is somewhat under-predicted by ISORROPIA, which is likely driven by the majority of  being in the particle phase and may be complicated by the difficulty of measuring HNO_3_ (Figure 3j). Still, *m*, *b*, and *R*^2^ are close to 1.0, 0.0, and 1.0 for the remaining species (Figure 3f–i). Setting the indoor RH to 98% (RH_room,opt_) in the ISORROPIA model drives semivolatile species to the particle phase, improving modeled-measured agreement. Similar behavior could occur at other RH input values when combined with lower temperature inputs, as shown in the optimized environmental condition results (Section S2). This outcome may suggest that the assumption of thermodynamic equilibrium, made by ISORROPIA, may not apply to this indoor setting. For instance, the high air exchange rate may have reduced the residence time of aerosols in the weight room, preventing them from ever reaching thermodynamic equilibrium. Furthermore, ISORROPIA is run under rather exotic conditions in this modeling scenario, where typical uses of ISORROPIA apply it to outdoor conditions. Alternatively, this outcome may indicate that there is a condensation driver that is not included in our model but has not been uncovered in this work.

Among many more potential explanations, some hypotheses are initially proposed for this observed need for a larger aerosol liquid water content for better modeled-measured agreement. These hypotheses include hysteresis, HVAC impacts, or a combination of the two; both pathways are difficult to test from the data and experimental design of the ATHLETIC campaign. For instance, setting the RH to a high value may reflect the possible history of the particles that deliquesced outdoors or in the HVAC system before coming into the weight room. Despite no active cooling (Figure S3) and the outdoor RH (RH_out_) not consistently being high (Figure S2), other *T* and RH extremes were thought to exist in parts of the HVAC zone that were not explicitly measured but could be significant. However, according to CU’s building management, only minimal heating occurred in the facility during the ATHLETIC campaign. Therefore, this hypothesis was deemed unlikely.

Furthermore, complexities in measurements were also considered as contributing to inaccuracies in standalone ISORROPIA simulated concentrations. Specifically, gases prone to partitioning to surfaces, like HNO_3_, may be reduced during the sampling process, so the measured HNO_3_ may under-represent what exists in the room air. However, increasing the HNO_3_ concentration to account for inlet losses of HNO_3_ led to a rejection of this possibility since the agreement of ISORROPIA-partitioned concentrations with measurements did not improve (Figures S6 and S7). Therefore, measurement uncertainties are not obviously causing inaccuracies in standalone ISORROPIA simulated concentrations.

Additionally, organic aerosol (OA) has relatively no impact on the total ALW needed to explain these observations, as shown in Figure S8 of the SI. To summarize, the OA ALW was found for the ATHLETIC observations using κOA parametrization from Rickards et al.,^57^ where κ is a single hygroscopicity parameter that describes the degree of hygroscopic growth for an aerosol component. Figure S8 shows that OA ALW is negligible compared to IA ALW. Therefore, omitting OA ALW is not to be blamed for the partitioning discrepancies. Thus, why ISORROPIA requires more water to be driven to the particle to perform well in the setting of the ATHLETIC campaign remains an open question.

Poor agreement at RH_room,meas_ is consistent with previous outdoor modeling campaigns when modeling species at RH values below 20%. For instance, Guo et al.^46^ had discarded data where the RH was below 20% since ISORROPIA is problematic in this RH range. For instance, at these low RH ranges, the activity coefficients associated with these highly concentrated solutions are uncertain, resulting in uncertainties in ISORROPIA’s pH predictions.^46^ Colorado occupies an “arid” climate zone,^58^ where indoor RH tends be especially low in colder months.^28^ Accordingly, the measured room RH is often below 20% (Figure S2). Thus, this poor agreement could result from ISORROPIA’s low trustworthiness in this RH domain. Although some data points fall on the one-to-one line in Figure 3b,c, ISORROPIA predicts an unrealistically high pH for these values (Figures S9 and S10). Using RH_room,opt_ instead puts the pH in a more realistic indoor range of ∼3 (Figures S11 and S12).^22^

### 3.2 Emissions and Deposition Relationship with Occupancy

**Figure 4:**
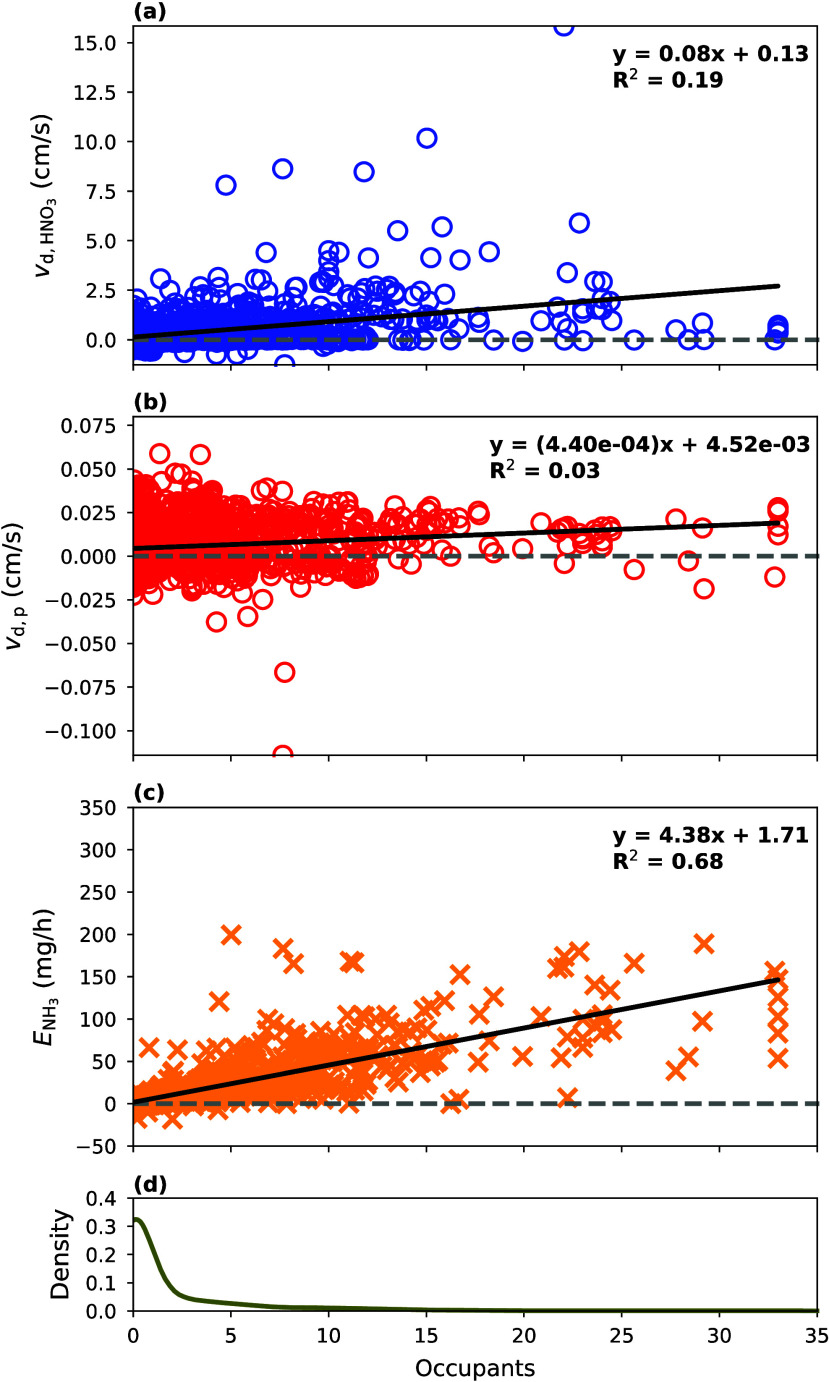
Linear relationships relating the number of occupants to  (a),  (b), and  (c), and a probability density function (d) shows the distribution of occupancy. The best fit line (black line), best-fit equation, and *R*^2^ value are displayed in each plot (a–c).

Linear relationships of , , and  to occupancy (Figure 4) and ΔCO_2_ (Figure S15) were derived to constrain those inputs for forward run IMAGES simulations. Occupancy was weakly correlated with  (*R*^2^ = 0.19) and  (*R*^2^ = 0.03). The  had a stronger correlation with occupancy than  did possibly because of the affinity of HNO_3_ for the water in human sweat. For instance, HNO_3_ may be more likely to deposit onto people with sweat on them than without. Still, occupancy does not seem to appreciably influence these deposition velocities given the small slope of their fits (*m* = 0.08 for  with respect to occupancy and *m* = 4.4 × 10^–4^ for  with respect to occupancy). The *y*-intercept depicted in Figure 4a,b is the deposition velocity of HNO_3_ and particles onto surfaces in the weight room without occupants present. The standard error for these slopes are shown in Table S3.

These deposition values might be weakly correlated to occupancy because the generally small number of people in the gym may not increase the total surface area by a large extent relative to the area without occupants. Manuja et al.^54^ suggest that only the first few largest items in the room contribute significantly to the total surface area. For instance, using the formula described in Dubois and Dubois^59^ to estimate body surface area (BSA) given body weight and height, the average adult American male (∼175 cm, ∼91 kg according to the Centers for Disease Control and Prevention) has a BSA of ∼2.07 m^2^. Assuming all occupants have about the same BSA, the 35 occupants would contribute to less than 2% of the total surface area (using *V* = 1700 m^3^ and  = 2.5 m^–1^) in the gym. Further, the order of magnitude of the near-constant  here aligns with previous indoor measurement campaigns such as Xu et al.^60^ and Offerman et al.^61^ Similarly, the range of  here (0.13–2.93 ) agrees with indoor measurements from Salmon et al.^62^ (0.24–1.34 ) and estimates from Lunden et al.^63^ (0.56 ).

Results demonstrate that occupancy is relatively well correlated with the net  (*R*^2^ = 0.68; Figure 4c).  being correlated with occupancy agrees with the findings of previous indoor field campaigns.^14,64^ For instance, the per person  computed here as the slope of the linear fit (∼4.38 ) is on par with  estimated from past studies (Furukawa et al.:^64^ ∼5.9  and Li et al.:^19^ 0.4–5.2 ). When no occupants are present, NH_3_ emissions still occur from building materials, and the *y*-intercept displayed in Figure 4c could represent the net NH_3_ emission rate attributed to the building source.^13^ However, this value is hard to compare to  from previous studies since it depends on multiple building parameters such as *T*, RH, and the air exchange rate.^13^ In the next set of IMAGES runs, the observed number of occupants in a room were used in the best-fit equations, displayed in Figure 4 (and Figure S15 when given ΔCO_2_), to estimate  and  at each time step within IMAGES. However, since the  value was near 0, a constant value of 0.0054 , taken from the average computed , was used instead.

### 3.3 IMAGES Evaluation

Room concentrations of inorganic particle and gas species were simulated using IMAGES for the ATHLETIC campaign based on measured supply airstream concentrations and room conditions, and the computed room concentration results were evaluated against room measurements. The degree to which RH_room,opt_ influenced IMAGES results was again assessed by running IMAGES with RH_room,meas_ and RH_room,opt_. The results presented here use the occupancy-based relationships of  and , and a constant  to determine deposition and emission parameters as described in Section 2.5. Results using the ΔCO_2_-based relationships instead are shown in Section S4.

**Figure 5:**
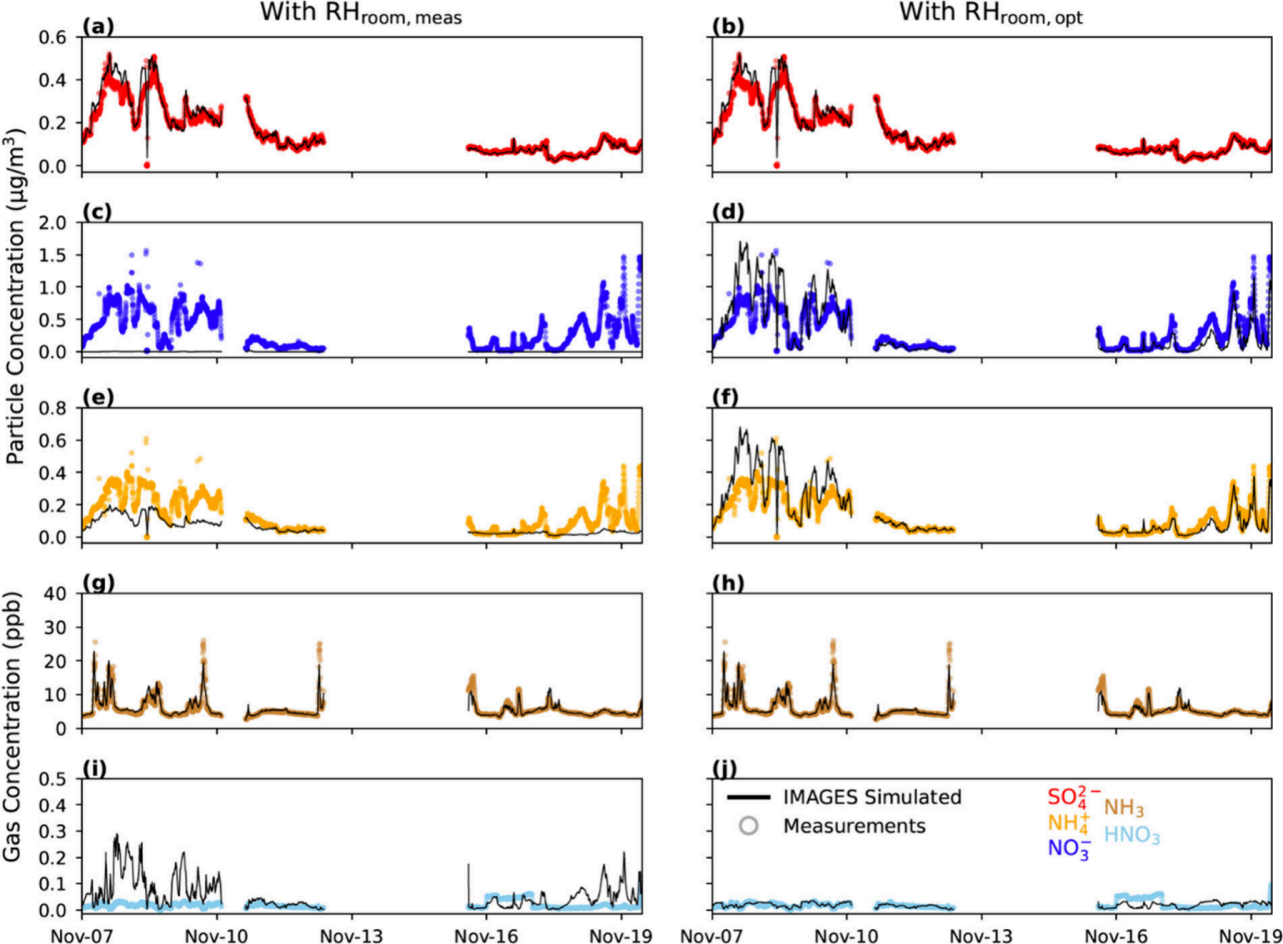
Time series of IMAGES simulated (solid lines) particle and gas concentrations using RH_room,meas_ (left column) and RH_room,opt_ (right column). Measured concentrations (circle markers) are shown for comparison.

**Figure 6:**
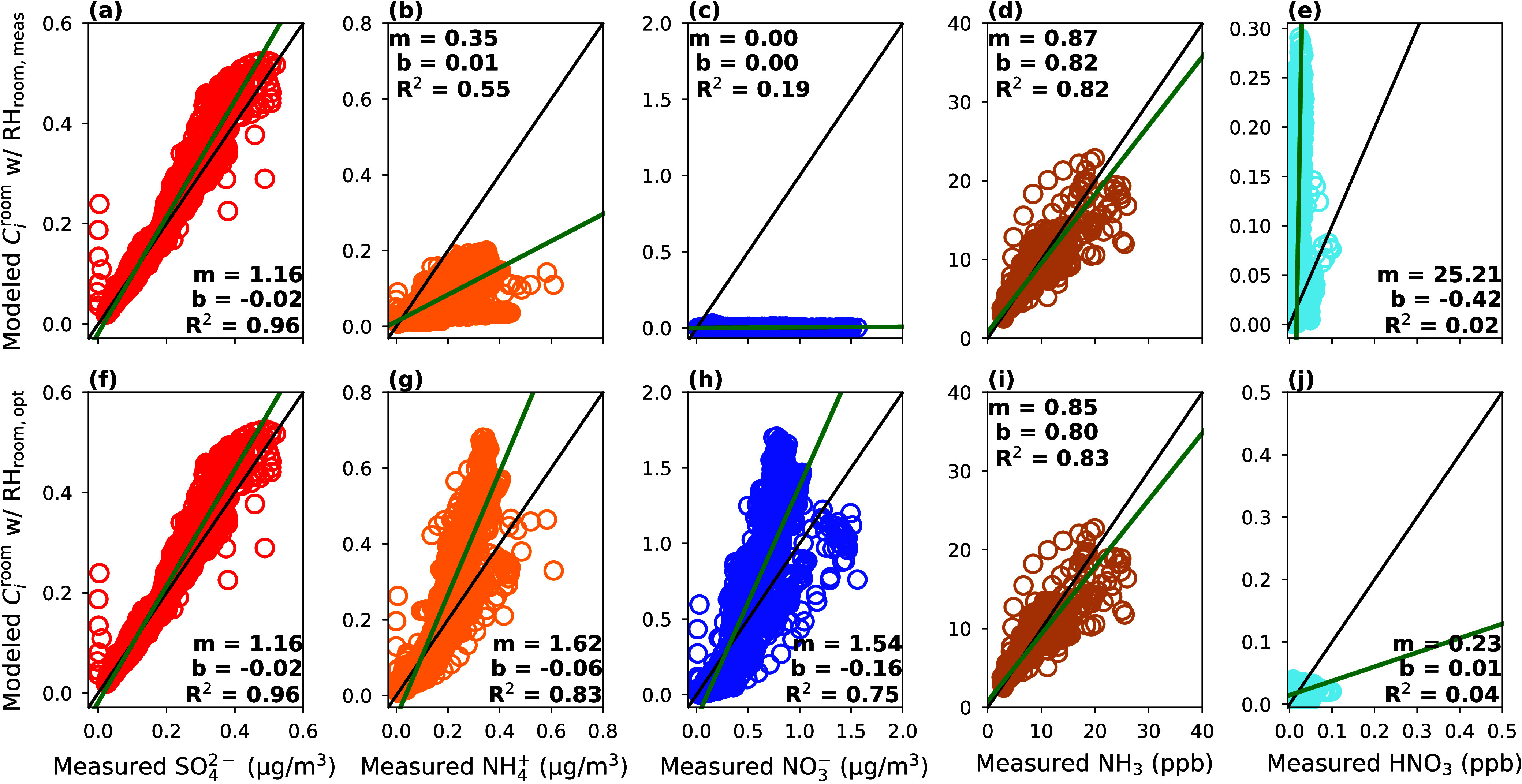
Comparison of IMAGES simulated and measured concentrations using RH_room,meas_ (a–e) and RH_room,opt_ (f–j) as model inputs. The green line represents the line of best fit calculated with an orthogonal regression, while the black line is the 1:1 line. The correlation coefficient, *R*^2^; slope, *m*; and *y*-intercept, *b*, are displayed for each regression.

The agreement of IMAGES results with measured room concentrations was strongly driven by the indoor RH used in the simulations and whether ISORROPIA well-predicted IA partitioning. For instance, when running IMAGES at measured *T* and RH conditions, IMAGES underestimates  and always allocates nitrate to the gas phase, but simulates  and NH_3_ well (Figure 5 left column and Figure 6a–e). Conversely, running IMAGES with RH_room,opt_ improves the agreement of modeled and measured concentrations of , HNO_3_, and . For instance, the IMAGES timeseries of HNO_3_ more closely follows HNO_3_ measurements when using RH_room,opt_ (Figure 5, right column) rather than measured , which it follows when using RH_room,meas_ (Figure 5 left column). This outcome was expected since similar results were presented in Section 3.1, in which the applicability of ISORROPIA in the weight room was evaluated independently.

After running IMAGES with the optimized RH_room,opt_ of 98% to correct the observed partitioning error associated with using ISORROPIA in this indoor setting,  and  are slightly overpredicted (*m* = 1.62 and 1.54, respectively), and NH_3_ and HNO_3_ are a bit underpredicted (*m* = 0.85 and 0.23, respectively). The poor HNO_3_ agreement may be driven by its almost negligible concentration. The fact that ,  and  occupancy-based estimations were used at each time step may explain these discrepancies between measurements and simulations, since the amount of mass in each phase may deviate from the measurements if the deposition and emission rates were inconsistent with those in reality. Although the actual emission and deposition rates at every step could have been used to produce more accurate results, estimating , , and  with occupancy (or ΔCO_2_) data is more valuable since they can be applied to future modeling domains. Still, IMAGES simulations predict IA concentrations well when using the occupant-based relationships with RH_room,opt_, but poorly estimates them when using RH_room,meas_. Similar results for the ΔCO_2_-based relationships are shown in Section S4. This result suggests that the deviation from the predicted equilibrium is the primary factor affecting the IMAGES performance when the RH is not optimized rather than the occupancy-based emission and deposition trends.

Still, a sensitivity analysis where  and  were varied was performed. For this sensitivity test,  was set to either 0 , 0.0058  (the average of the trend line in Figure 4b), or 0.03  (the 95th percentile of the trend line in Figure 4b). Additionally,  was set to either 0 , 0.28  (the average of the trend line in Figure 4a), or 1.22  (the 95th percentile of the trend line in Figure 4a). Results from this sensitivity analysis are shown in Figures S20 and S21 of the SI. To summarize, the model was not sensitive to changes in particle deposition. However, omitting  returned a portion of data points where the modeled semivolatile particle species concentrations agreed with measurements, which corresponded to the cases when ISORROPIA estimated unrealistically high pH values. Increasing  eliminated any . Additionally, a sensitivity analysis was conducted where *A*/*V* was set to 0.5 m^–1^, 2.5 m^–1^, or 10 m^–1^. These results show that the modeled-measured agreement did not improve by increasing *A*/*V*. However, lowering *A*/*V* returned some data points where the modeled semivolatile particle species matched measured concentrations. However, the cases where good agreement occurred were due to ISORROPIA estimating unrealistically high pH values (as discussed in Section 3.1). Results from this sensitivity test can be found in Figure S22 of the SI.

## 4 Conclusions

The thermodynamic inorganic aerosol model ISORROPIA that was recently integrated into our comprehensive indoor aerosol model, IMAGES, was applied here to simulate the partitioning of inorganic particle- and gas-phase species in a weight room with occupants during the ATHLETIC indoor measurement campaign. The measurements in this campaign provided the first opportunity to evaluate the performance of ISORROPIA indoors. Linear relationships, which related , , and  to occupancy, were derived from measurements since these parameters were unknown but were required for indoor modeling with IMAGES.  correlated strongly with occupancy, but  and  did not since the occupants contributed little to the total surface area in the gym. Still, the range of estimated , , and  agreed well with values from previous studies.  and  correlations and a constant  were used during the indoor modeling to parametrize deposition and emission rates using the observed occupancy at the model time steps.

IMAGES only performed well when the aerosol liquid water content in ISORROPIA was made greater than measured indoor environmental conditions of air temperature and RH would suggest. The necessary increase of the indoor RH to a higher RH_room,opt_ was determined in an independent parametric analysis since *T* was relatively constant throughout the campaign. ISORROPIA not accounting for hysteresis effects was hypothesized to play a role in why ISORROPIA-partitioned concentrations agreed with measurements when the measured RH was used. However, this hypothesis, as well as inlet losses of HNO_3_ contributing to the poor agreement were deemed unlikely. Other possible explanations could be evaluated in the future, such as the building walls being more complex than this model assumes, as they may act as a source or sink of inorganic species depending on conditions. Estimating deposition and emission rates also contributes to the slight overpredictions of particles and underpredictions of gases. Ultimately, IMAGES simulations predicted indoor IA concentrations in a gym with people with good agreement with measurements when RH was optimized with observations.

Therefore, with IMAGES, detailed modeling can be performed to understand better how aerosols’ physical state and composition changes, such as when transported from the supply duct to the room. Knowing the physical state and composition of contaminants is crucial. For example, aerosol composition influences their physicochemical properties, such as volatility, hygroscopicity, and density, which affect aerosol behavior. However, an increased RH (or decreased *T*) was required to obtain accurate inorganic partitioning for this modeling scenerio, Depending on why changes in RH or *T* were needed here, this fix may or may not work in future modeling scenerios. Therefore, future work will build upon this study by looking into ISORROPIA’s indoor partitioning error. Additionally, an air handling unit (AHU) module will be developed, enabling researchers to simulate how aerosols’ physical state and composition alter during outdoor-to-indoor transport.

## Supporting Information Available

The SI starts with a note on how the ATHLETIC Campaign data was preprocessed for use with IMAGES. Then, the SI gives more information about how ISORROPIA performed indoors by (1) describing in detail the parametric test that was initially discussed in Section 2.2, (2) analyzing the various hypotheses made for why ISORROPIA needs a high RH or low temperature to produce accurate gas-particle partitioning, and (3) showing how ISORROPIA performed indoors when it is run using the stable mode. Next, the SI describes how we determined that running ISORROPIA with an RH of 98% would produce the most accurate gas-particle partitioning results. Then, emission and deposition rate trends as they relate to ΔCO_2_ are shown. Additionally, how outliers were found from these emission and deposition trends is described. Finally, IMAGES is run and evaluated using the ΔCO_2_-based deposition and emission trends (PDF)
