# Supplementary material for: Modeling Indoor Inorganic Aerosol Concentrations During the ATHLETIC Campaign with IMAGES
Source: ACS EST Air. 2024 Sep 3;1(9):1084–95. doi: 10.1021/acsestair.4c00060 (PMC11406535; doi:10.1021/acsestair.4c00060)
Supplement: Supplementary file 1 — ea4c00060_si_001.pdf [file ea4c00060_si_001.pdf]

Supplemental Information for:

Modeling Indoor Inorganic Aerosol Concentrations during the ATHLETIC Campaign with IMAGES

Bryan Berman[1], Bryan Cummings[1], Hongyu Guo[2], Pedro Campuzano-Jost[2], Jose Jimenez[2], Demetrios Pagonis[3], Douglas Day[2], Zachary Finewax[2], Anne Handschy[2], Benjamin A. Nault[4], Peter DeCarlo[5], Shannon Capps[1], Michael Waring[1,*]

[1]Department of Civil, Architectural and Environmental Engineering, Drexel University, Philadelphia, PA, 19104

[2]Department of Chemistry and Cooperative Institute for Research in Environmental Sciences (CIRES), University of Colorado at Boulder, Boulder, CO 80309, USA

[3]Department of Chemistry and Biochemistry, Weber State University, Ogden, UT 84408, USA

[4]Center for Aerosol and Cloud Chemistry, Aerodyne Research, Inc., Billerica, MA, 01821, USA

[5]Department of Environmental Health and Engineering, Johns Hopkins University, Baltimore, MD 21218, USA

[*]*Corresponding author email: msw59@drexel.edu*

Table of Contents

S1. Note on preprocessing ATHLETIC Campaign data 3

S2. ISORROPIA's performance indoors 3

S3. Optimizing indoor environmental conditions.. 15

S4. Relating emissions and deposition rates to ΔCO_2.. 18

S5. IMAGES evaluation 24

S1. Note on preprocessing ATHLETIC Campaign data

During the ATHLETIC campaign, instruments switched between sampling the room and supply air every 5-10 minutes via an automated valve system.[1,2] However, IMAGES requires continuous inputs over consistent timesteps. Therefore, all measured data (such as room and supply duct concentrations, RH, supply *T*, and occupancy) were linearly interpolated over 5-min intervals before being used as inputs to IMAGES. NH_3 and HNO_3 were measured less frequently in the duct due to sampling concerns. Thus, three input datasets for the IMAGES framework were created and run separately. These datasets' date/time stamps are as follows: 1) Nov. 7 00:33 – Nov. 10 02:53, 2) Nov. 10 15:48 – Nov. 12 09:28, and Nov. 15 14:48 – Nov. 19 11:33.

S2. ISORROPIA's performance indoors

As discussed in Section 2.2, a parametric test was performed to determine the specific *T* and RH combinations that produce the necessary thermodynamic conditions within ISORROPIA to obtain reasonable agreement with measurements. Specifically, the parameters *T* and RH were varied from 260 to 300 K at intervals of 1 K and 1 to 100% at intervals of 1%, respectively. This parametric test was done to determine if certain *T* and RH combinations yielded ISORROPIA-partitioned concentrations to be in better agreement with ATHLETIC study measurements (Figure S1). Then, the coefficient of determination (R^2), slope (*m*), and y-intercept (*b*) of the measured and ISORROPIA partitioning fraction in the weight room, $\varepsilon_{i,\mathrm{room}}$, were compared for each *T* and RH combination using an orthogonal regression since there is uncertainty in both modeled and measured data (Figure S1). Figure S1 shows heatmaps of the coefficient of determination (R^2) (a, b), slope (m) (c, d), and y-intercept (b) (c, d) from the lines of best fit of ISORROPIA's predicted $\varepsilon_{i,\mathrm{room}}$ vs. the measured $\varepsilon_{i,\mathrm{room}}$. The areas of best performance are highlighted by the lightest color in the heatmaps shown in Figure S1. Although it remains unclear why agreement improves under these specific environmental conditions, hysteresis and

inlet losses of HNO_3 during measurements were hypothesized to be potential pieces to the puzzle and explored.

We first explored the hypothesis that not accounting for hysteresis effects contributes to ISORROPIA not agreeing with measurements. Figure S2 shows a time series of the different temperatures measured at various parts of the HVAC system and the fraction of outdoor air that enters the mixing box during the ATHLETIC campaign. The similar mixed T (T_{mixed}), discharge T ($T_{\text{discharge}}$), and supply duct T (T_{supply}) suggests there was at most slight heating (and no cooling). Instead, the outdoor T closely following the outdoor air fraction implies economizing was done to achieve a fairly constant T_{mixed}, and the target $T_{\text{discharge}}$ of ~287-288 K at this main air handling unit (AHU). Prior to the air delivery back to the weight room, the air passes through local variable air volume (VAV) terminal boxes that can provide additional conditioning where likely some heating occurred at times (and/or in transit within ducts). To our knowledge, no humidification processes occurred in the HVAC. Nevertheless, it was thought that particles may have retained their aqueous phase as they were brought indoors. However, since the outdoor RH (RH_{out}) is not consistently high, this theory was rejected (Figure S2). Still, an unaccounted-for condensation sink may have been present.[3] Inputting $RH_{\text{room, meas}}$ to ISORROPIA results in the nitrate being simulated as a gas rather than a particle most of the time (Figure S3). Lowering the room T or increasing the RH seems to make up for the unaccounted-for condensation sink, despite us not being unable to identify it. Additionally, using ISORROPIA's stable mode, which allows particles to be aqueous or solids, showed no improvement and resulted in a worse agreement (Figures S4 and S5) than when run with the metastable mode.

The second hypothesis for why ISORROPIA's predicted $\varepsilon_{i,\text{room}}$ disagreed with measurements was because of the complexities associated with measuring sticky gases such as HNO_3. ISORROPIA was run (and $\varepsilon_{i,\text{room}}$ was calculated) with an increased amount of HNO_3 added to the original measured value (up to ten times as much) to make up for the potential HNO_3 loss. However, Figures S6 and S7 show that no improvements are made when HNO_3 is increased; thus, this hypothesis was rejected.

In Figure S8, the OA ALW was found using κOA parameterization from Rickards *et al.* Figure S8 shows that OA ALW is on the order of about 0.05 - 0.1 ug sm^{-3}. Based on the total inorganic mass and their κ (~ 3-6x larger than kappa OA), the increase in IA ALW (caused by adjusting the RH upwards) is on the order of 5-15 ug sm^{-3}. Therefore, not accounting for OA (in this data set) has no impact on the total ALW needed to explain the observations.

Despite generally having poor modeled vs. measured agreement, some instances exist where NO_3^- and NH_4^+ are in good agreement. Figures S9 and S10 show one-to-one plots of measured vs. ISORROPIA simulated NO_3^- and NH_4^+ with $RH_{room,meas}$ and are colored by pH. When the concentrations are in good agreement, the pH, an output of ISORROPIA, is unrealistically high, with some values reaching ~15. This result shows that the model does not perform well, even when modeled concentrations appear to match measurements when using $RH_{room,meas}$. However, when $RH_{room,opt}$ is used for the IMAGES simulations, the pH is consistently around a value we would expect indoors (Figure S11 and S12).[4]

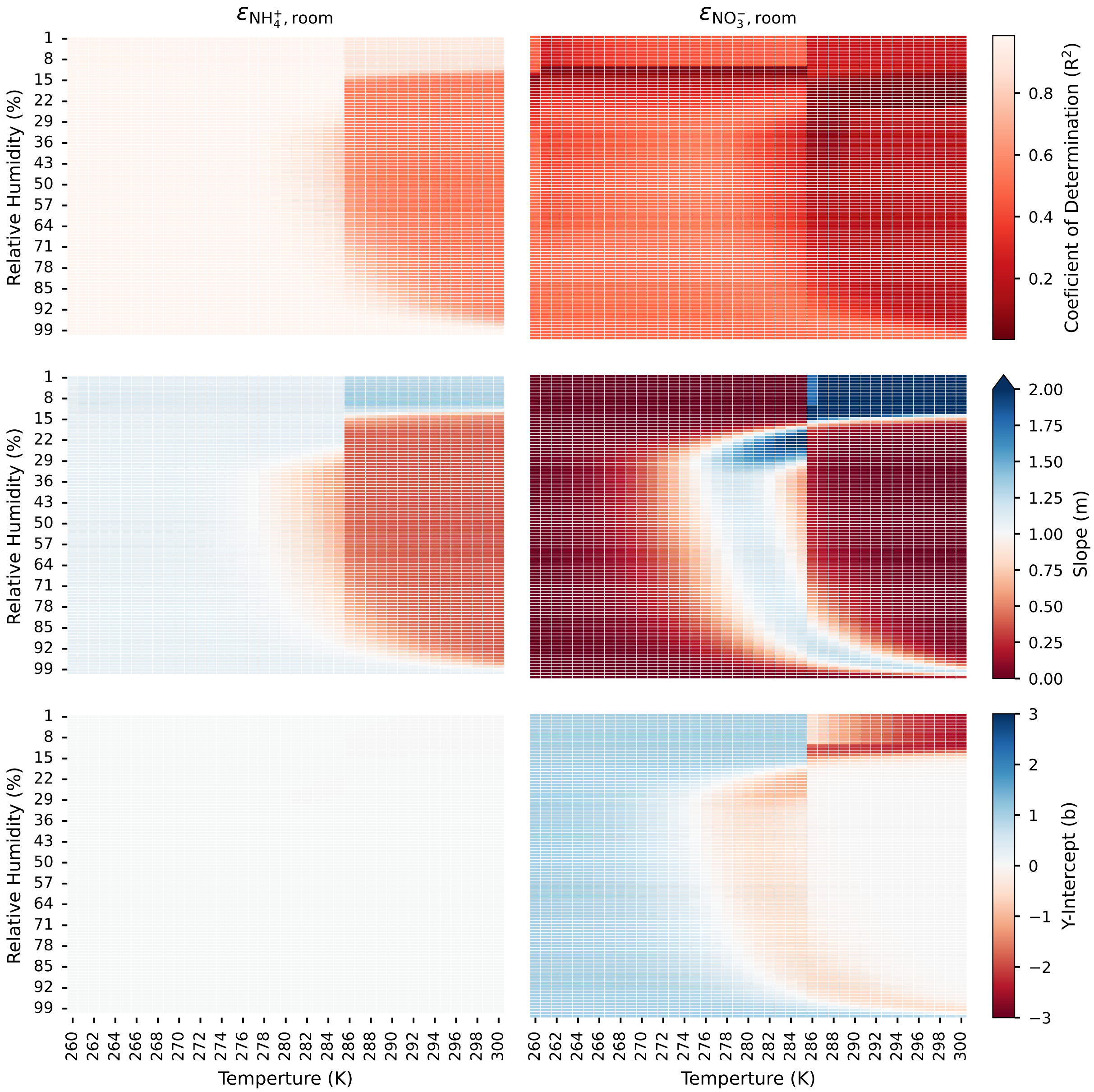

Figure S1. Heatmaps of R^2 (a, b), m (c, d), and b (c, d) from the lines of best fit of ISORROPIA $\varepsilon_{i,\mathrm{room}}$ vs. measured $\varepsilon_{i,\mathrm{room}}$. Each cell represents a T and RH case that was inputted into ISORROPIA.

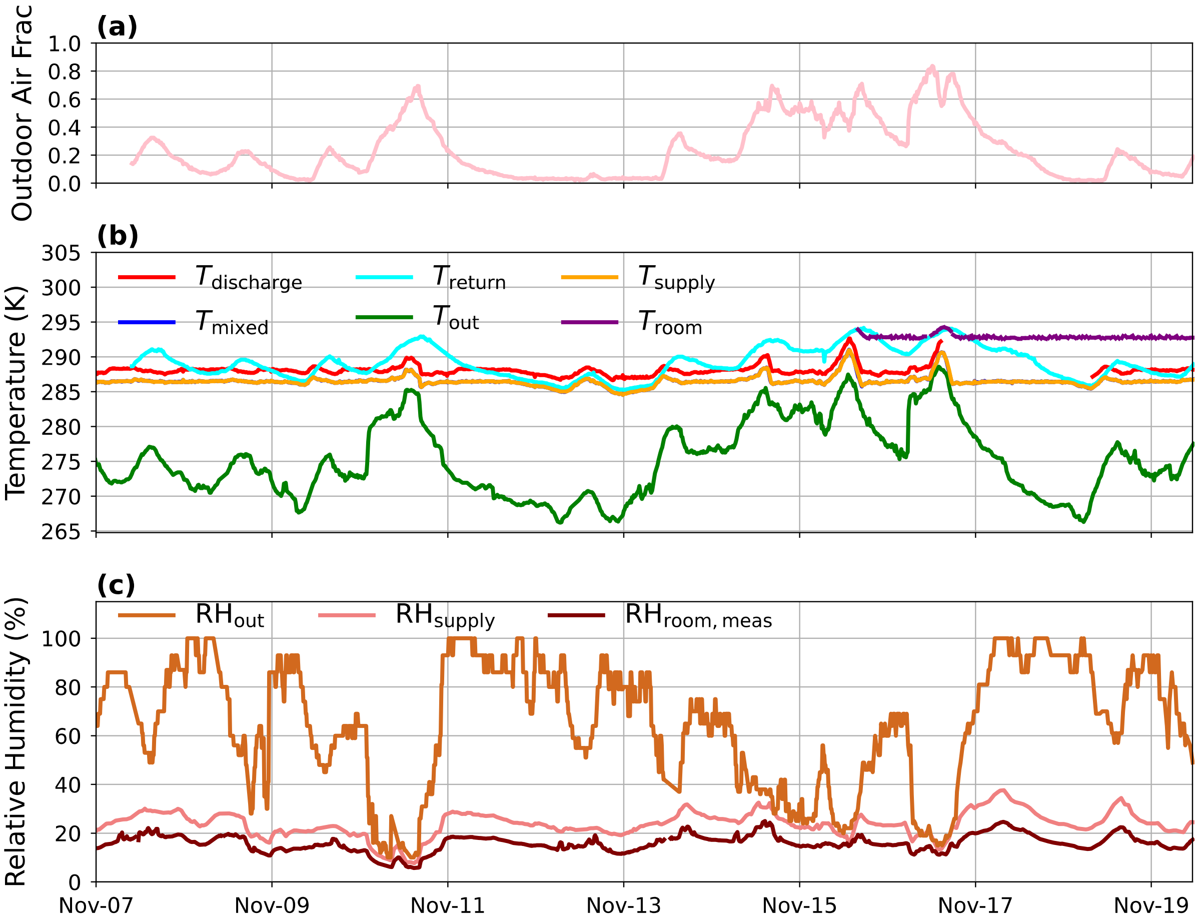

Figure S2. Timeseries of the fraction of outdoor air that enters the mixing box (a), the different temperatures ($T_{discharge}$, T_{mixed}, T_{return}, T_{out}, T_{supply}, and T_{room}) (b), and RHs (RH_{out}, RH_{supply}, $RH_{room,meas}$) (c) measured during the ATHLETIC campaign. The outdoor RH was obtained from NOAA.

93

94

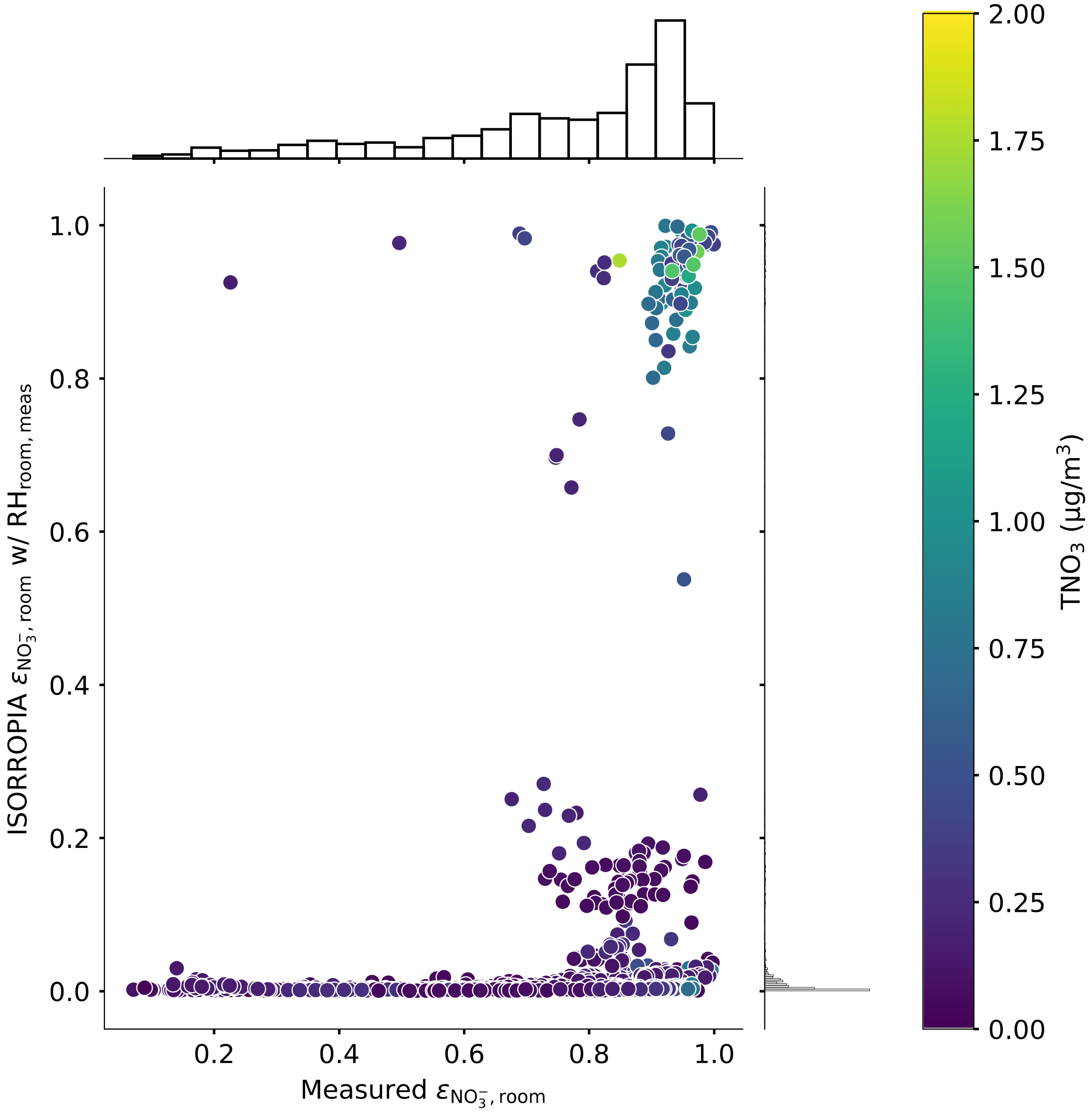

Figure S3. Comparison of ISORROPIA simulated (using $RH_{room,meas}$) and measured $\varepsilon_{i,room}$. The markers are colored by the amount of total nitrate ($TNO_3 = NO_3^- + HNO_3$). The histograms on the top and right side of the plot show the distribution of data points for standalone ISORROPIA simulated results and measurements, respectively.

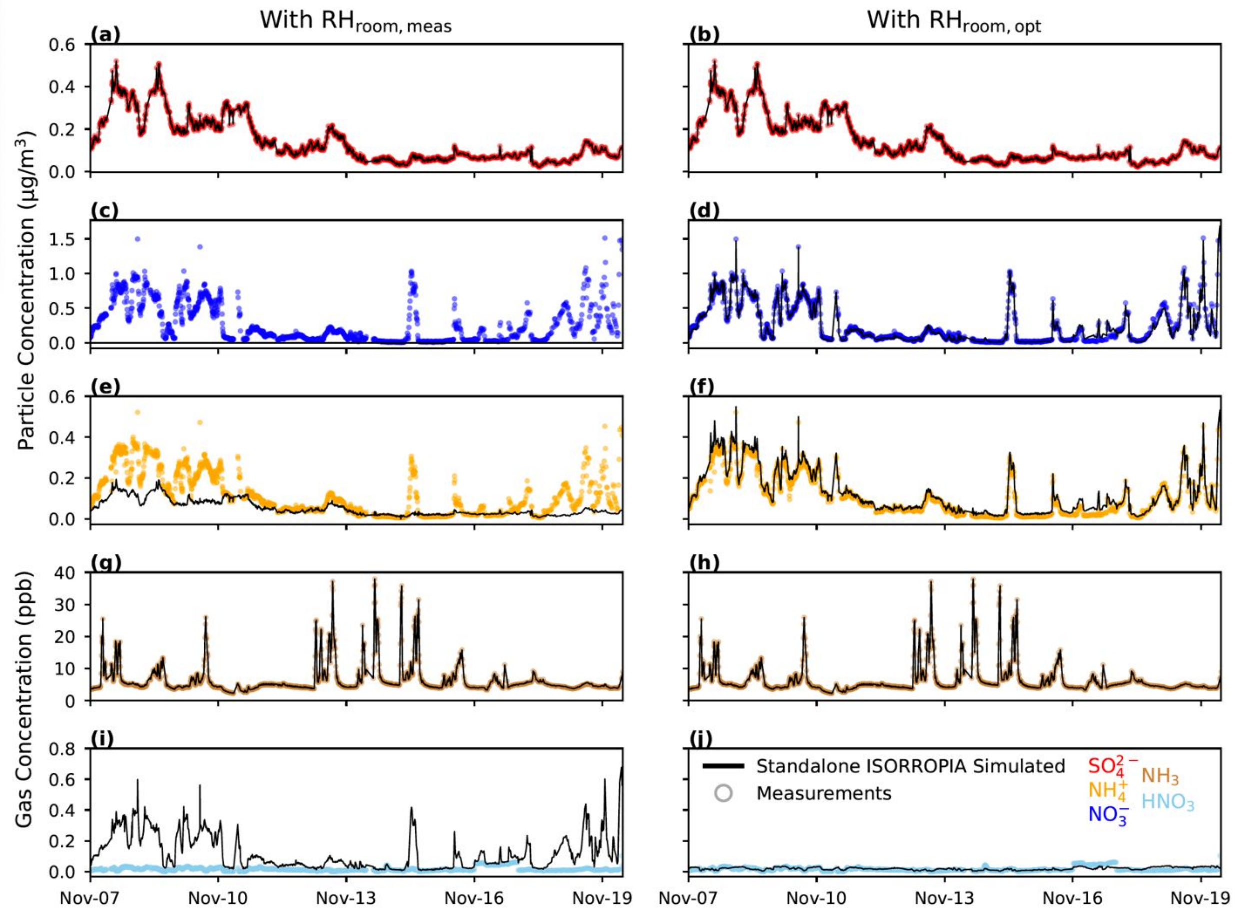

Figure S4. Timeseries of standalone ISORROPIA simulated (black line) particle and gas concentrations using $RH_{room,meas}$ (left column) and $RH_{room,opt}$ (right column). Measured concentrations (open circles) are shown for comparison. ISORROPIA was run in the stable mode here.

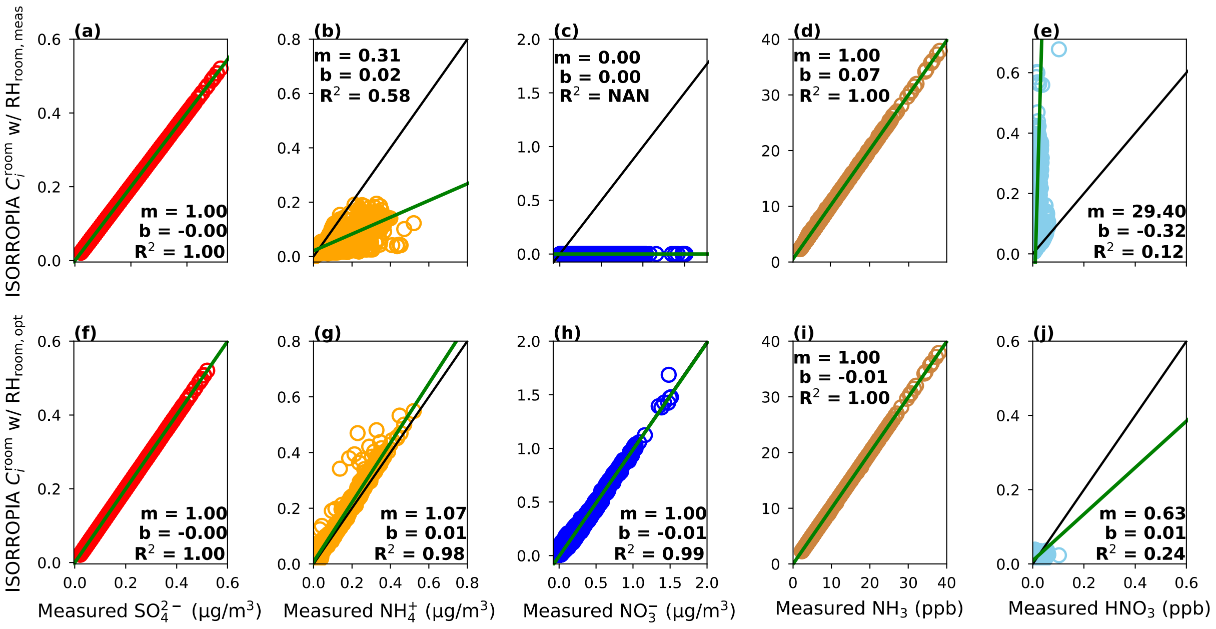

Figure S5. Comparison of ISORROPIA-partitioned concentrations against measured concentrations using $RH_{room,meas}$ (a-e) and $RH_{room,opt}$ (f-j) as inputs to ISORROPIA. The green line represents the line of best fit calculated with an orthogonal regression, while the black line is the 1:1 line. The correlation coefficient, R^2; slope, *m*; and *y*-intercept, *b*, are displayed for each regression. ISORROPIA was run in the stable mode here.

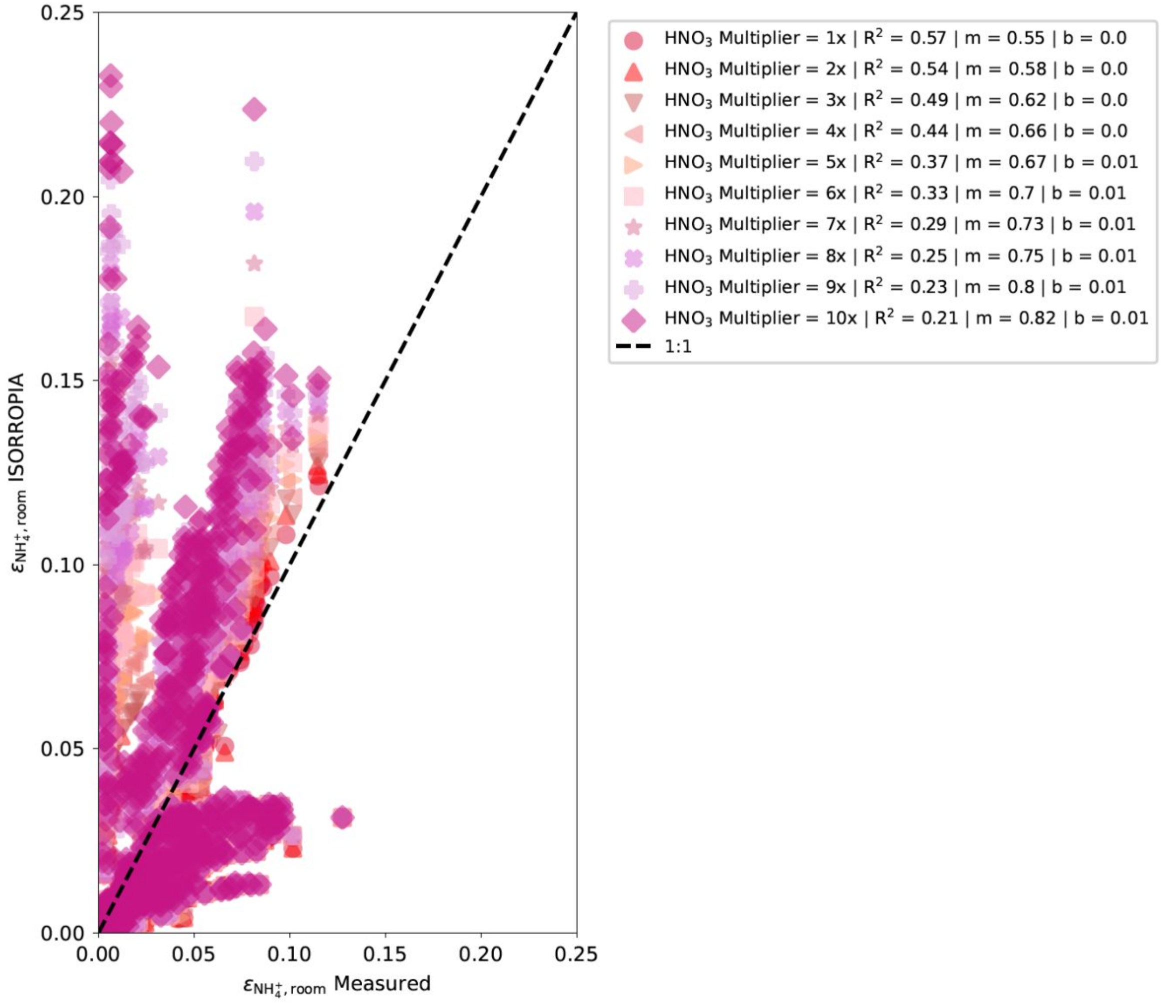

Figure S6. A comparison of ISORROPIA vs. measured $\varepsilon_{NH_4^+,room}$ best fit lines for each increased HNO_3 case. The amount HNO_3 was increased by was ranged from no increase (HNO_3 Multiplier = 1x) to ten times the original amount (HNO_3 Multiplier = 10x) and is listed on the right-side table. The table also includes the line of best-fit statistics, which are R^2, m, and b.

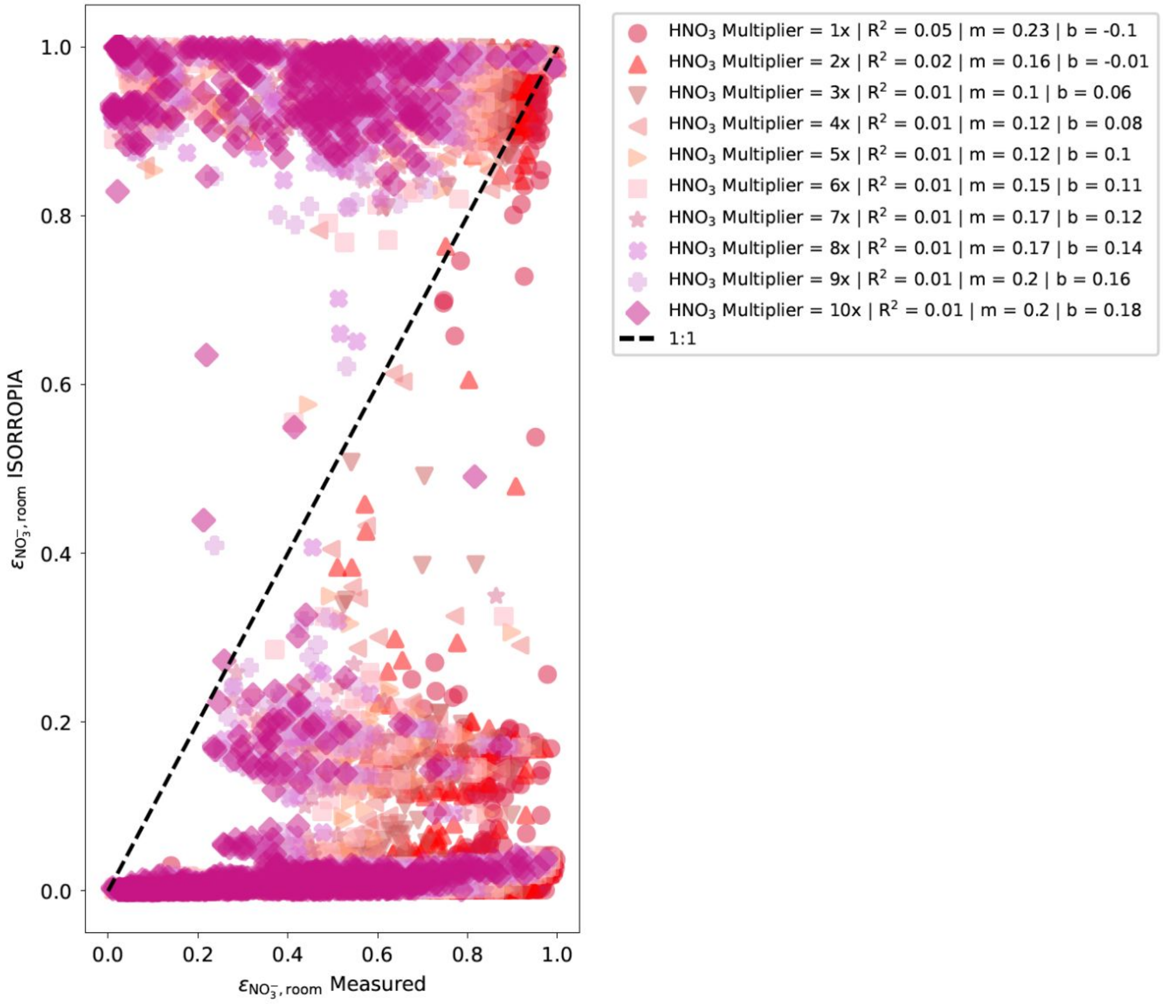

Figure S7. A comparison of standalone ISORROPIA simulated vs. measured $\varepsilon_{NO_3^-,room}$ best fit lines for each increased HNO_3 case. The amount HNO_3 was increased by was ranged from no increase (HNO_3 Multiplier = 1x) toten times the original amount (HNO_3 Multiplier = 10x10) and is listed on the right-side table. The table also includes the line of best-fit statistics, which are R^2, *m*, and *b*.

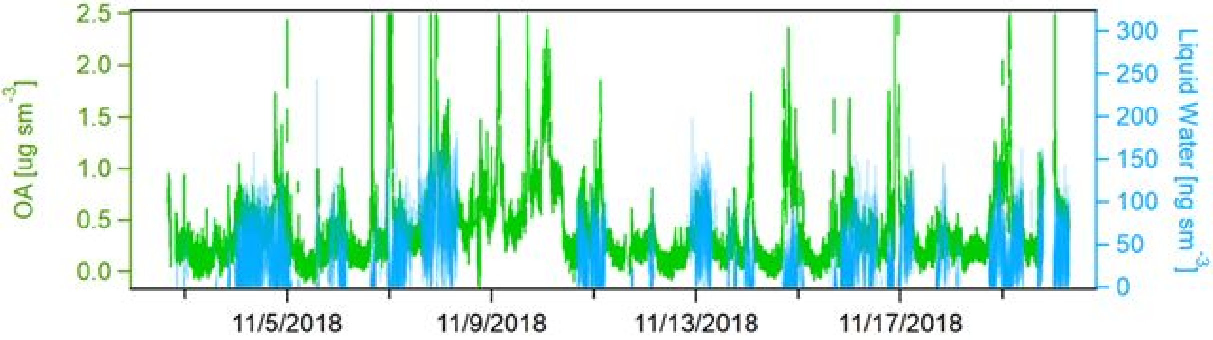

Figure S8. Time series of OA mass on left y-axis and OA ALW on right y-axis (calculated using Rickards *et al.*[5]) This data assumes a constant RH of 75%.

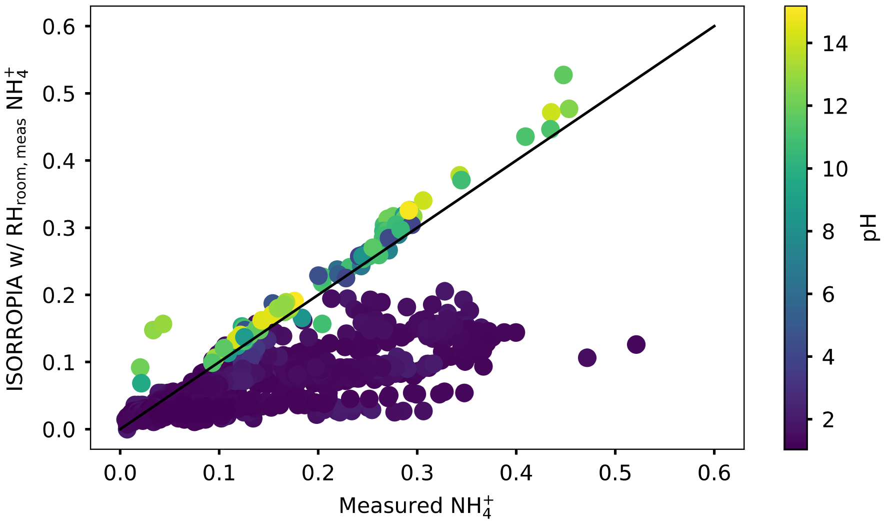

Figure S9. One-to-one plot comparing standalone ISORROPIA simulated NH_4^+, using $RH_{room,meas}$, against measured NH_4^+. Data points are colored by the pH calculated by ISORROPIA.

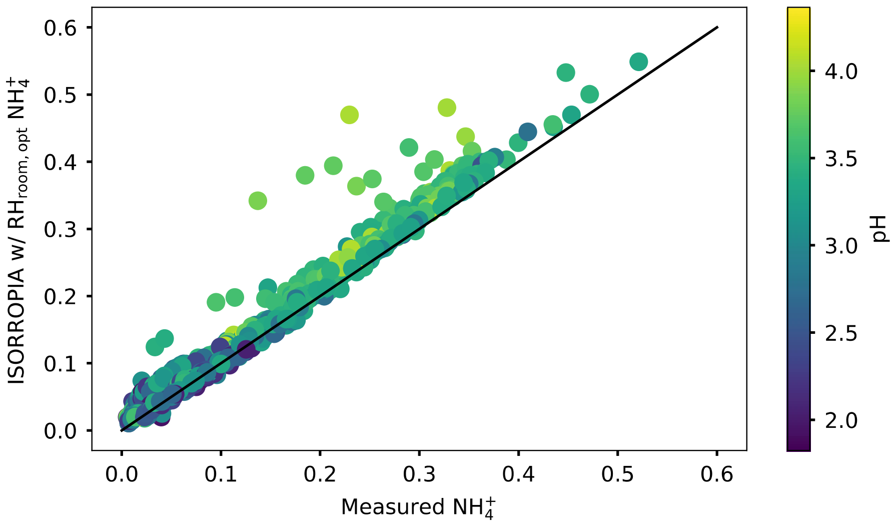

Figure S10. One-to-one plot comparing standalone ISORROPIA simulated NH_4^+, using $RH_{room,opt}$, against measured NH_4^+. Data points are colored by the pH calculated by ISORROPIA.

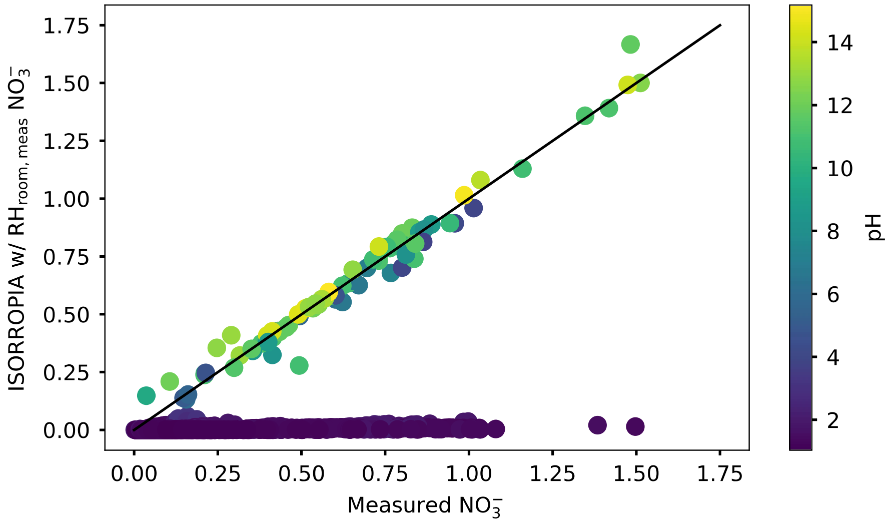

Figure S11. One-to-one plot comparing standalone ISORROPIA simulated NO_3^-, using $RH_{room,meas}$, against measured NO_3^-. Data points are colored by the pH calculated by ISORROPIA. A pH above 5 in the absence of refractory species is considered unrealistic.[6]

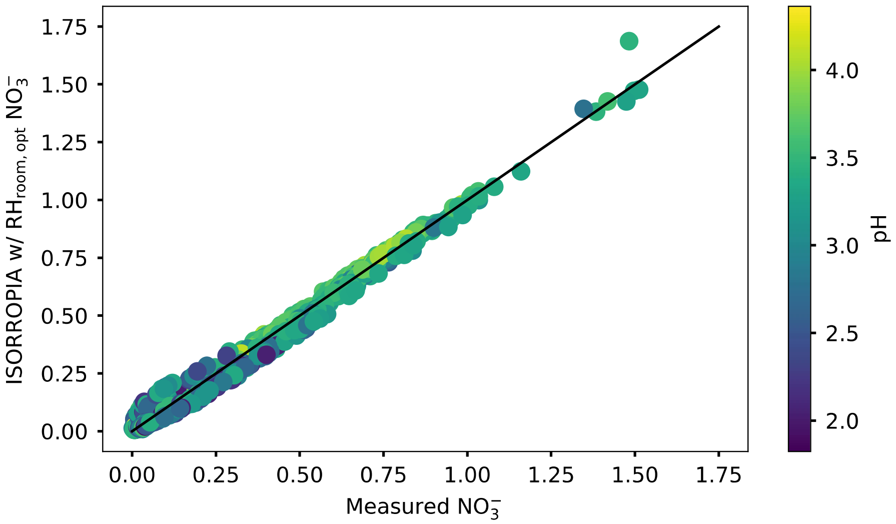

Figure S12. One-to-one plot comparing standalone ISORROPIA simulated NO_3^-, using $RH_{room,opt}$, against measured NO_3^-. Data points are colored by the pH calculated by ISORROPIA.

S3. Optimizing indoor environmental conditions

The environmental conditions that minimized ISORROPIA's partitioning error were picked based on the minimum distance between a perfect one-to-one correlation and the actual correlation of ISORROPIA vs. measured $\underline{\varepsilon_{room}}$ (described in Section 2.3). Figure S13 shows that the distance was smallest when the T was low or RH was high. However, since T_{room} was controlled at ~293 K throughout the ATHLETIC campaign, a single optimized RH value ($RH_{room,opt}$) was chosen at 293 K. Figure S14 shows just the 293 K column from Figure S13. The smallest distance in the 293 K column (Figure S14) occurred at RH = 98%, and thus, $RH_{room,opt}$ = 98%. Optimizing the RH at T = 293 K gives a similarly exceptional partitioning agreement as optimizing the T and RH across the full range of values. For instance, the distance at T = 293 K and RH = 98% is 0.25. When considering the full range of environmental conditions, the smallest distance is 0.24, which occurs at T = 274 K and RH = 45%. Since these distances are almost the same, the T = 293 K and RH = 98% condition was chosen to preserve the room temperature measurements.

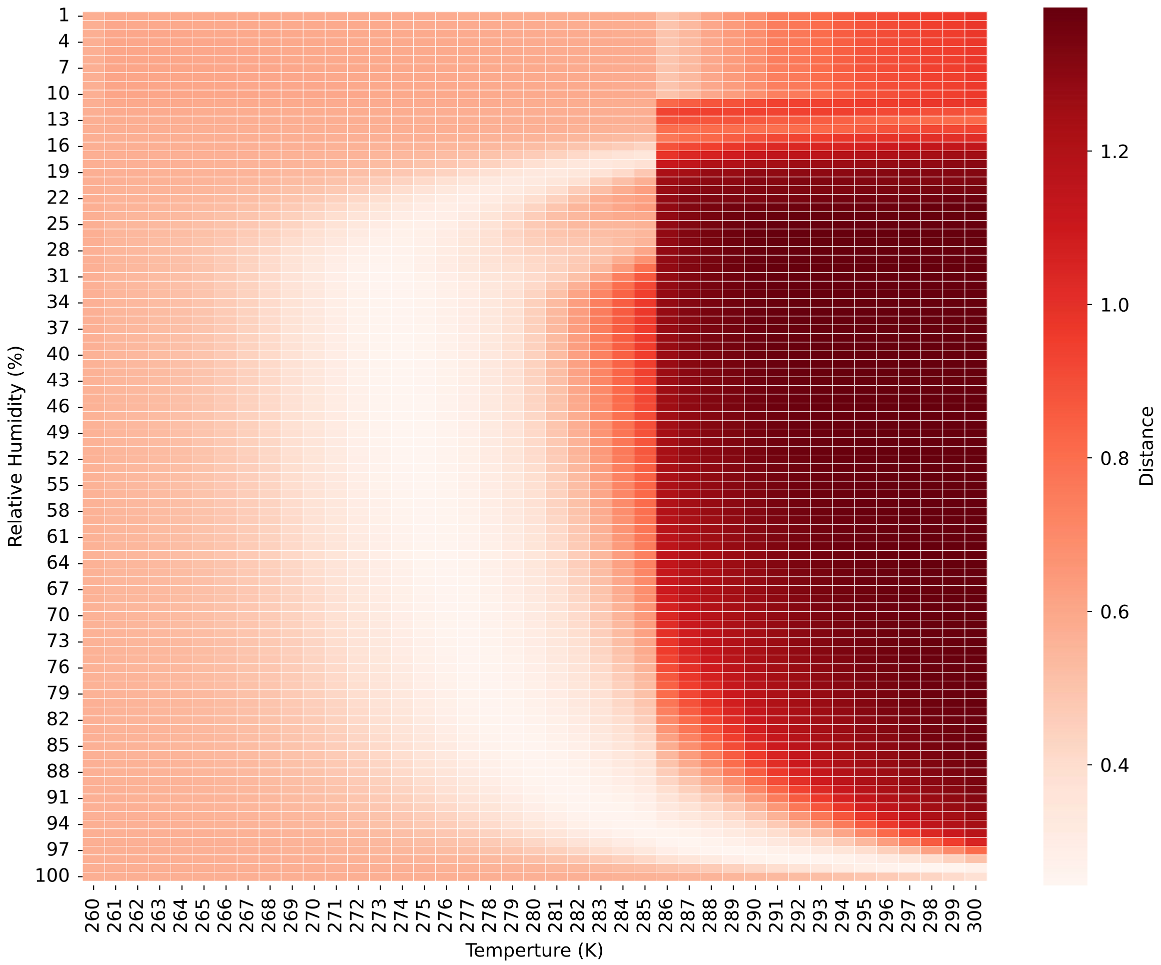

Figure S13. Heatmap of the distance between a perfect one-to-one correlation and the actual correlation of $\underline{\boldsymbol{\varepsilon}_{\mathbf{room}}}$. Each cell represents a T and RH case where $\underline{\boldsymbol{\varepsilon}_{\mathbf{room}}}$was calculated, and the modeled-measured line of best-fit statistics was compared against perfect one-to-one best-fit statistics.

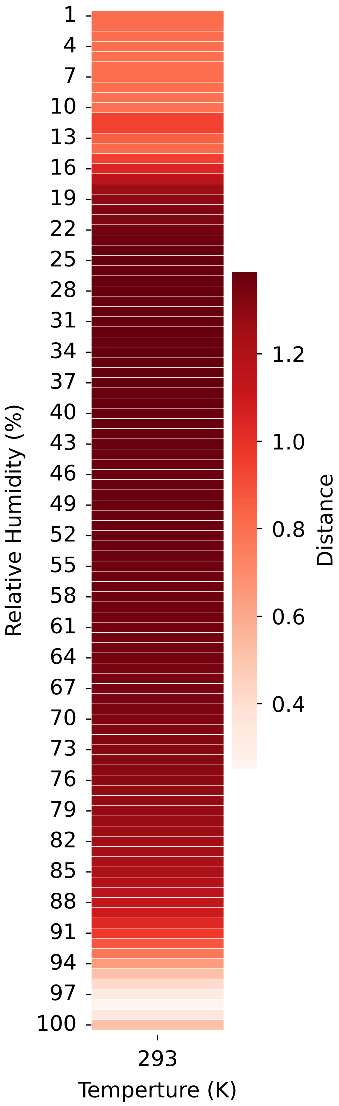

Figure S14. The distance between a perfect one-to-one correlation and the actual correlation of $\underline{\boldsymbol{\varepsilon}_{\mathbf{room}}}$ at 293 K.

S4. Relating emissions and deposition rates to ΔCO_2

ΔCO_2 can be used as an indicator of building occupancy when the number of occupants is not provided. Therefore, linear trends relating $v_{\mathbf{d,HNO_3}}$, $v_{\mathbf{d,\,p}}$, and $\boldsymbol{E}_{\mathbf{NH_3}}$ to $\mathbf{\Delta CO_2}$ (Figure S15) were derived using the same algorithm described in Section 2.5. $\mathbf{\Delta CO_2}$ denotes the difference between indoor and outdoor CO_2 concentrations. Since outdoor CO_2 was not measured, it was taken to be the 5th percentile of the indoor CO_2 concentration (~424.1 ppm). The analytical solution to Eq. 3 (Eq. S1) allows C_i to be approximated after a short period of time ($\boldsymbol{\delta^t}$, h):

$$C_i^{t+1} \cong C_i^t \exp(-l_i^t \delta^t) + \frac{S_i^t}{l_i^t}\left(1 - \exp\left(-l_i^t \delta^t\right)\right) \quad \text{(S1)}$$

By using $\boldsymbol{\lambda}_{\mathbf{supply}}$ and an N number of $\boldsymbol{C}_{\boldsymbol{i},\mathbf{supply}}$ measurements with $\boldsymbol{\varepsilon}_{\boldsymbol{i},\mathbf{room}}$ (which was computed by ISORROPIA), N-1 $\boldsymbol{E}_{\mathbf{NH_3}}$, $\boldsymbol{\beta}_{\mathbf{p}}$, and $\beta_{\mathbf{HNO_3}}$ vectors occurring between two adjacent time-series measurements were explicitly back-calculated from Eq. S1 using a built-in Python solver. The source and loss rates (Equations S2 – S7) were specific to the species considered when solving for each parameter. For instance, non-volatile $\mathbf{SO_4^{2-}}$, the species considered when solving for $\beta_{\mathbf{p}}$, does not have any gas-phase sources or losses indoors. Therefore, the source and loss rates can be defined as:

$$S_{\mathrm{TSO_4^{2-}}} = \lambda_{\mathrm{supply}} C_{\mathrm{TSO_4^{2-},supply}} C_{\mathrm{TSO_4^{2-},supply}} \quad \text{(S2)}$$

$$l_{\mathrm{TSO_4^{2-}}} = \lambda_{\mathrm{supply}} + \beta_{\mathrm{p}} \quad \text{(S3)}$$

NO_3^- and HNO_3 were the only species considered when solving for $\beta_{\mathrm{HNO_3}}$. Thus, the source and loss rates used here were:

$$S_{\mathrm{TNO_3^-}} = \lambda_{\mathrm{supply}} C_{\mathrm{TNO_3^-,supply}} C_{\mathrm{TNO_3^-,supply}} \quad \text{(S4)}$$

$$l_{\mathrm{TNO_3^-}} = \left(1 - \varepsilon_{\mathrm{TNO_3^-,room}}\right)(\lambda_{\mathrm{supply}} + \beta_{\mathrm{HNO_3}}) + \varepsilon_{\mathrm{TNO_3^-,room}} \varepsilon_{\mathrm{TNO_3^-,room}} (\lambda_{\mathrm{supply}} + \beta_{\mathrm{p}}) \quad \text{(S5)}$$

Similarly, NH_4^+ and NH_3 were the only species considered when solving for $E_{\mathrm{NH_3}}$, and so the source and loss rates for this case were:

$$S_{\mathrm{TNH}_4^+} = \lambda_{\mathrm{supply}} C_{\mathrm{TNH}_4^+,\mathrm{supply}} C_{\mathrm{TNH}_4^+,\mathrm{supply}} + \frac{E_{\mathrm{NH}_3}}{V} \tag{S6}$$

$$l_{\mathrm{TNH}_4^+} = \left(1 - \varepsilon_{\mathrm{TNH}_4^+,\mathrm{room}}\right)\lambda_{\mathrm{supply}} + \varepsilon_{\mathrm{TNH}_4^+,\mathrm{room}} \varepsilon_{\mathrm{TNH}_4^+,\mathrm{room}} (\lambda_{\mathrm{supply}} + \beta_{\mathrm{p}}) \tag{S7}$$

When a concentration was below the detection limit, data at that timestep and the timestep prior were not included in the linear regressions. Limits of detection for $\mathbf{SO_4^{2-}}$, $\mathbf{NH_4^+}$, and $\mathbf{NO_3^-}$ were determined using methods from Drewnick *et al.*[7] Limits of detection for $\mathbf{NH_3}$ and $\mathbf{HNO_3}$ were provided on the measurement instrument manufacturers' website.[8,9] Detection limit values are provided in Table 1. Standard errors for the slopes can be found in Table 2. Additionally, the trendlines pertaining to occupants and $\mathbf{\Delta CO_2}$ when no data points were removed from the regression are shown in Figures S16 and S17. Therefore, Figures S16 and S17 show the need for removing data points associated with concentrations that dip below the detection limit.

Table S1. Limits of Detection for SO_4^{2-}, NH_4^+, NO_3^-, NH_3 and HNO_3.

**Species**	**Limit of Detection ($\frac{\mu g}{m^3}$)**
SO_4^{2-}	0.0288
NH_4^+	0.0036
NO_3^-	0.0132
NH_3	0.004
HNO_3	0.0035

Table S2. Percentage of room measurements used to evaluate IMAGES simulation that fell below the limit of detection (LOD).

**Species**	**Percentage below LOD**
SO_4^{2-}	1.4%
NH_4^+	2.4%
NO_3^-	0%
NH_3	0%
HNO_3	0.64%

Table S3. Standard errors of slopes from linear regression relating that relate occupants or ΔCO_2 to v_{d,HNO_3}, $v_{d,\,p}$, and E_{NH_3}.

	Figure 4	Figure S15
v_{d,HNO_3}	0.0033	0.00031
$v_{d,\,p}$	5.4e-5	5.4e-6
$v_{d,\,p}$	0.006	0.0073

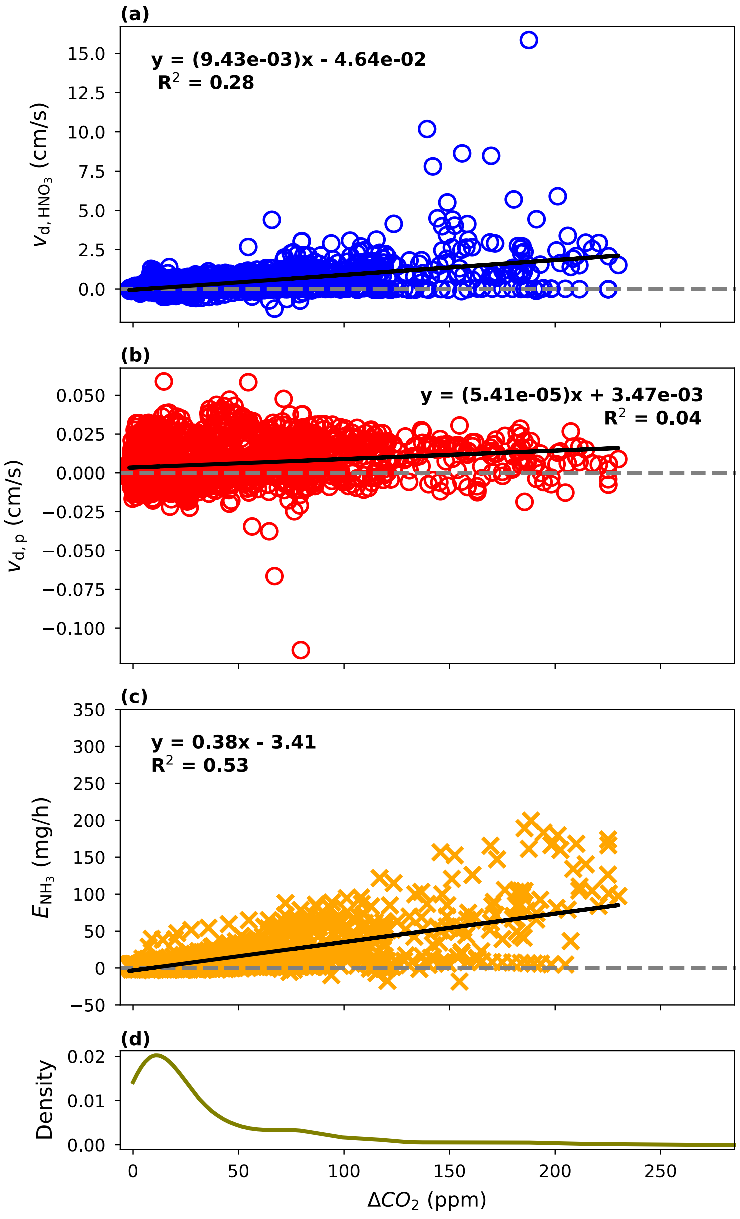

Figure S15. Linear trends relating $\boldsymbol{\Delta CO_2}$ to $\boldsymbol{v_{d,HNO_3}}$ (a), $\boldsymbol{v_{d,p}}$ (b), and $\boldsymbol{E_{NH_3}}$ (c). A probability density function (PDF) (d) is also displayed to show the distribution of data points related to $\boldsymbol{\Delta CO_2}$. The best fit line (black line), best-fit equations, and $\boldsymbol{R^2}$ value are displayed in each plot (a-c).

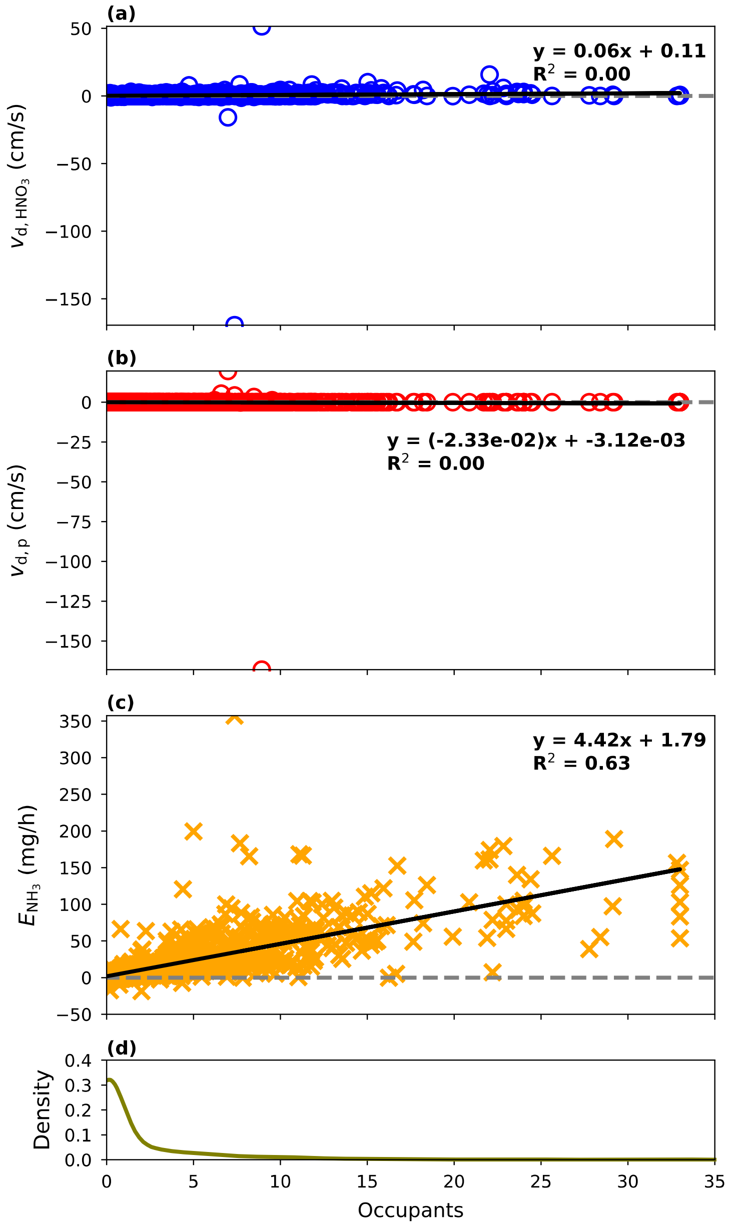

Figure S16. Linear trends relating occupants to $\boldsymbol{v_{d,HNO_3}}$ (a), $\boldsymbol{v_{d,p}}$ (b), and $\boldsymbol{E_{NH_3}}$ (c). A probability density function (PDF) (d) is also displayed to show the distribution of data points related to occupants. The best fit line (black line), best-fit equations, and $\boldsymbol{R^2}$ value are displayed in each plot (a-c). No data points were removed from the linear regressions here.

152

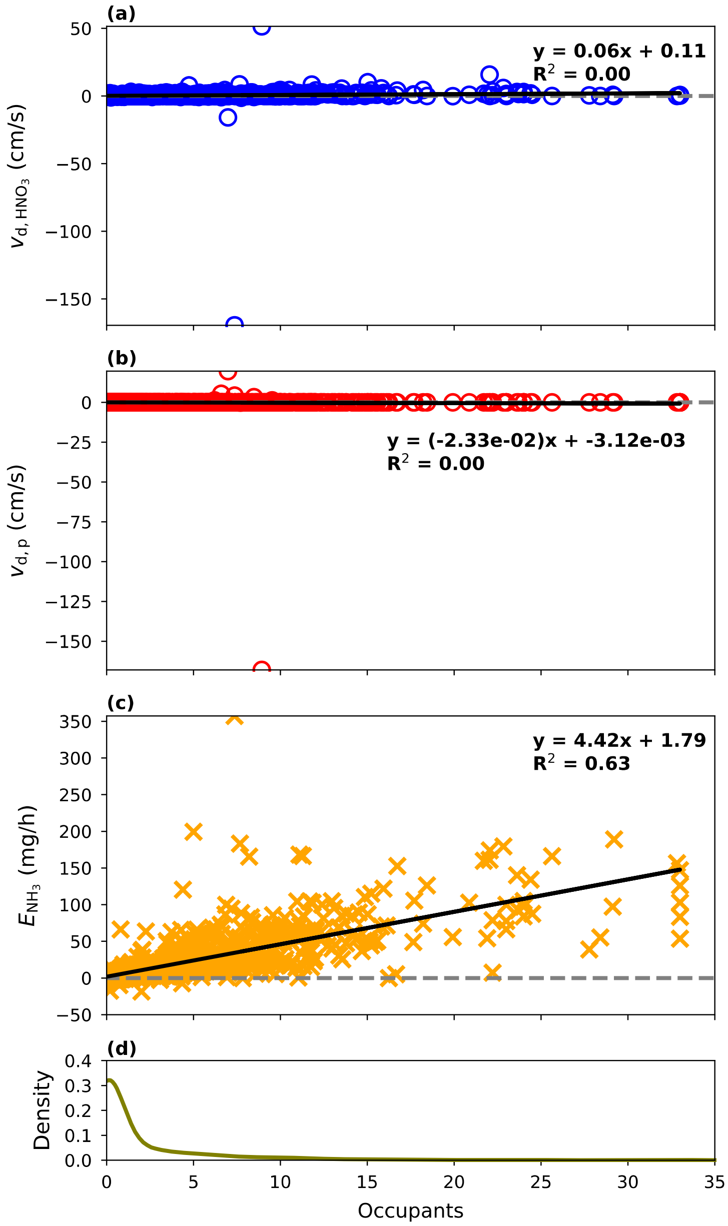

Figure S17. Linear trends relating $\Delta \mathbf{CO_2}$ to $\boldsymbol{v}_{\mathbf{d,HNO_3}}$ (a), $\boldsymbol{v}_{\mathbf{d,p}}$ (b), and $\boldsymbol{E}_{\mathbf{NH_3}}$ (c). A probability density function (PDF) (d) is also displayed to show the distribution of data points related to $\Delta \mathbf{CO_2}$. The best fit line (black line), best-fit equations, and $\boldsymbol{R}^2$ value are displayed in each plot (a-c). No data points were removed from the linear regressions here.

S5. IMAGES evaluation

IMAGES was run using the ΔCO_2-based deposition and emission trends described in Section S3. Figures S18 and S19 show similar timeseries and one-to-one comparison results to those presented in Section 3.3; thus, the analysis is similar. However, one distinct difference is that NH_3 has slightly worse modeled-measured agreement when using the ΔCO_2-based trends than when using the occupant-based trends. This may be because ΔCO_2 is not as accurate at indicating the level of occupancy in a building as physically counting each person. Despite this, IMAGES was still run with ΔCO_2-based trends since it is a more standard field measurement than counting occupants.

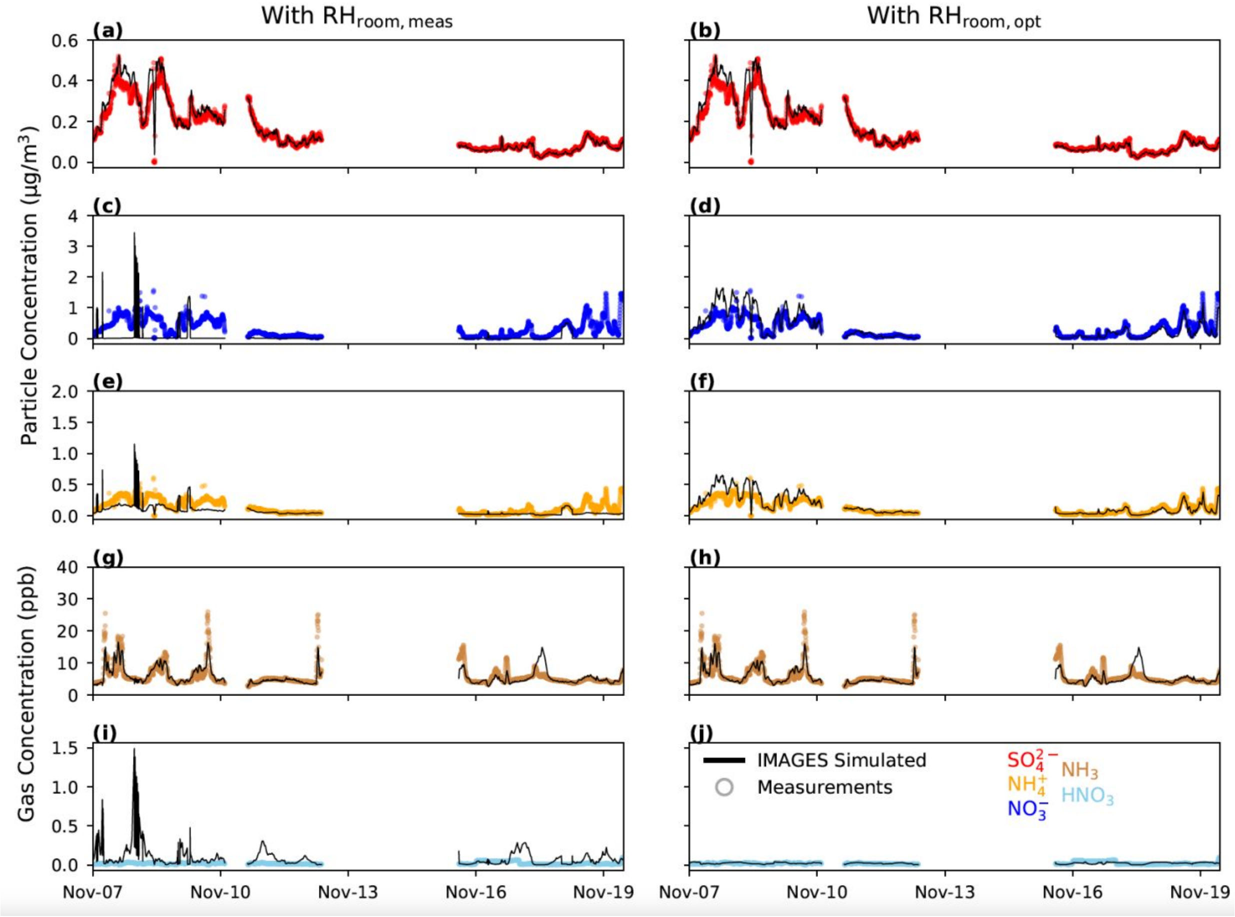

Figure S18. Timeseries of IMAGES simulated (black line) particle and gas concentrations using $\mathrm{RH_{room,meas}}$ (left column) and $\mathrm{RH_{room,opt}}$ (right column). ΔCO_2-based deposition and emission trends were utilized for IMAGES here. Measured concentrations (open circles) are shown for comparison.

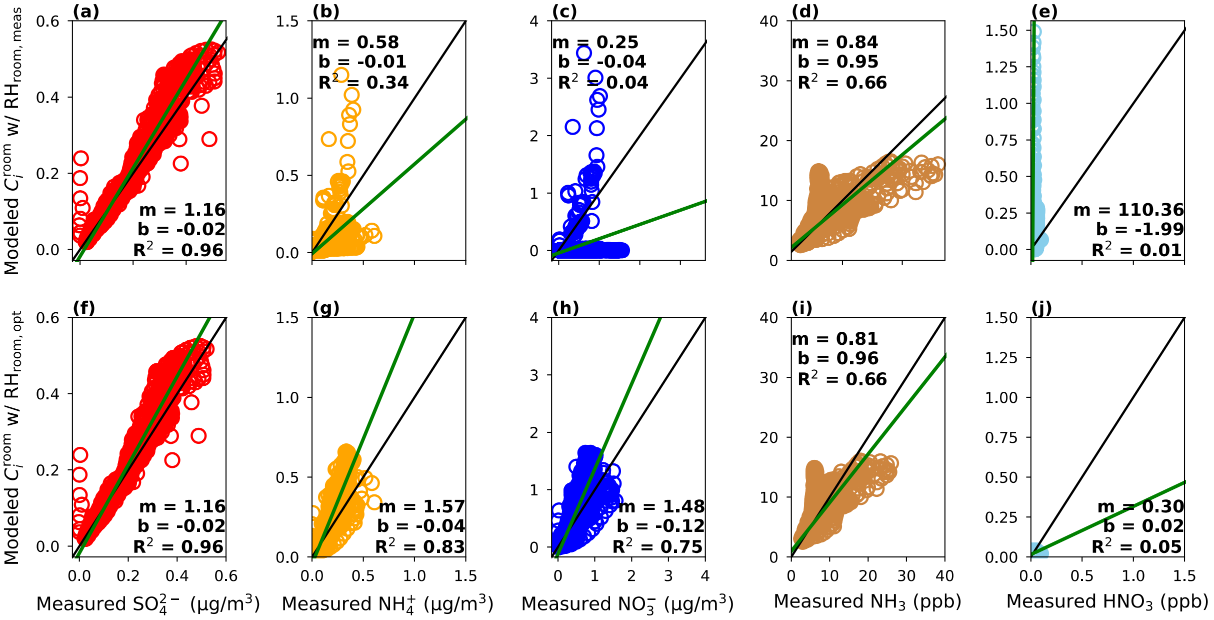

Figure S19. Comparison of IMAGES simulated and measured concentrations using $RH_{room,meas}$ (a-e) and $RH_{room,opt}$ (f-j) as model inputs. ΔCO_2-based deposition and emission trends were utilized for IMAGES here. The green line represents the line of best fit calculated with an orthogonal regression, while the black line is the 1:1 line. The correlation coefficient, R^2; slope, *m*; and *y*-intercept, *b*, are displayed for each regression.

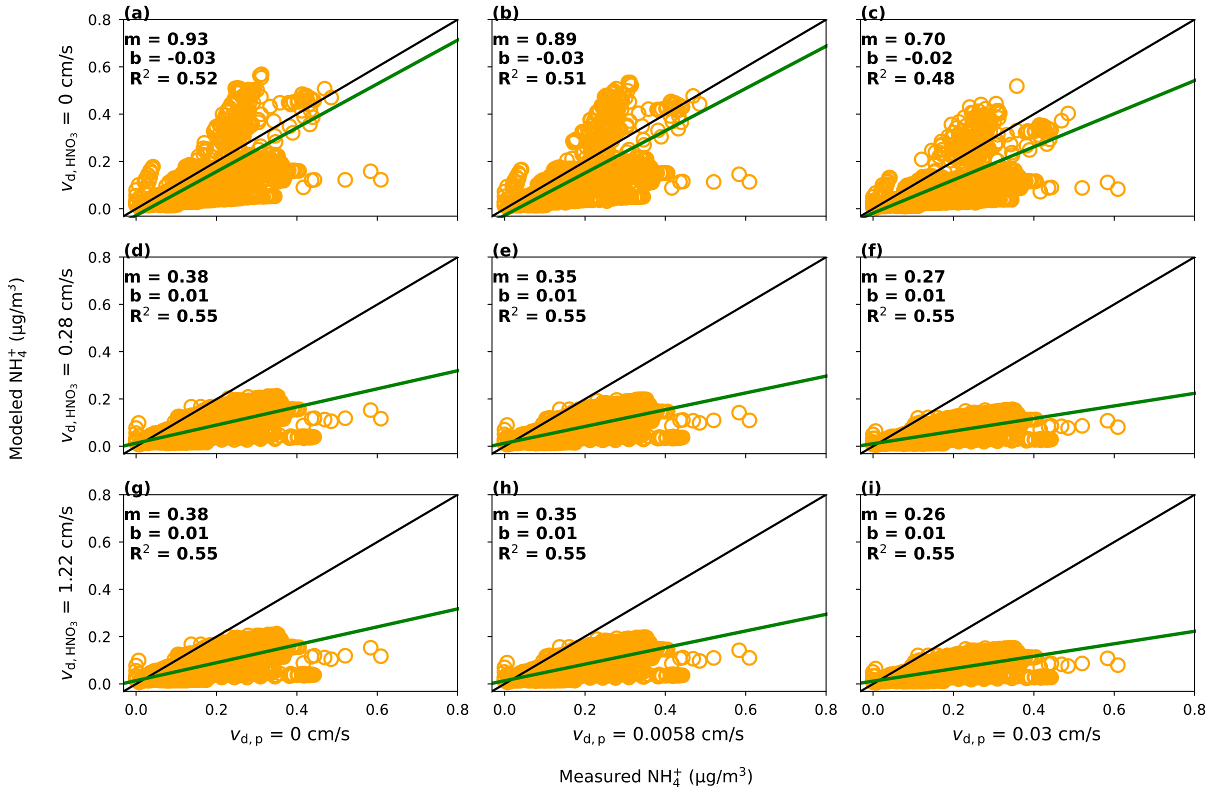

Figure S20. One-to-one plots comparing modeled NH_4^+ concentrations in the room to NH_4^+ concentration measurements. IMAGES was run with the measured temperature and RH conditions. $v_{\mathrm{d,p}}$ was set to either 0 $\frac{\mathrm{cm}}{\mathrm{s}}$ (left column), 0.0058 $\frac{\mathrm{cm}}{\mathrm{s}}$ (middle column), or 0.03 $\frac{\mathrm{cm}}{\mathrm{s}}$ (right column). Additionally, $v_{\mathrm{d,HNO_3}}$ was set to either 0 $\frac{\mathrm{cm}}{\mathrm{s}}$ (top row), 0.28 $\frac{\mathrm{cm}}{\mathrm{s}}$ (middle row), or 1.22 $\frac{\mathrm{cm}}{\mathrm{s}}$ (bottom row).

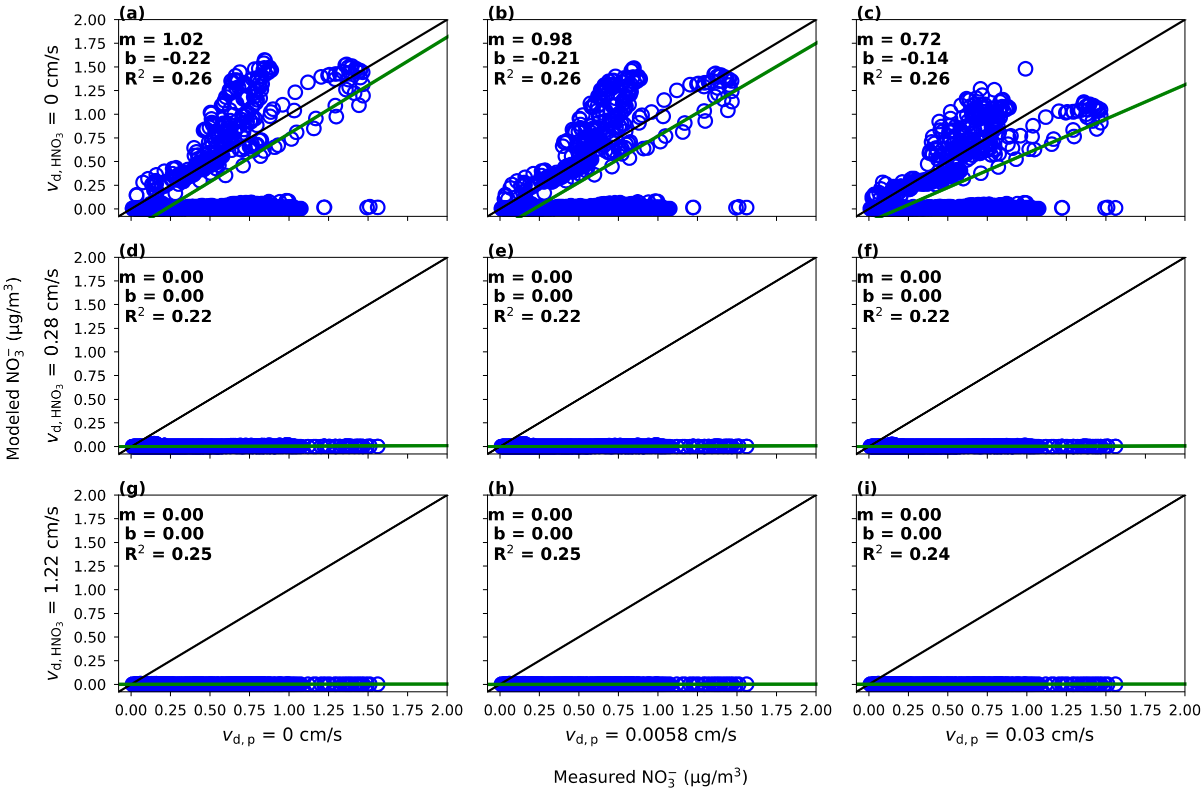

Figure S21. One-to-one plots comparing modeled NO_3^- concentrations in the room to NO_3^- concentration measurements. IMAGES was run with the measured temperature and RH conditions. $v_{d,p}$ was set to either 0 $\frac{cm}{s}$ (left column), 0.0058 $\frac{cm}{s}$ (middle column), or 0.03 $\frac{cm}{s}$ (right column). Additionally, v_{d,HNO_3} was set to either 0 $\frac{cm}{s}$ (top row), 0.28 $\frac{cm}{s}$ (middle row), or 1.22 $\frac{cm}{s}$ (bottom row).

168

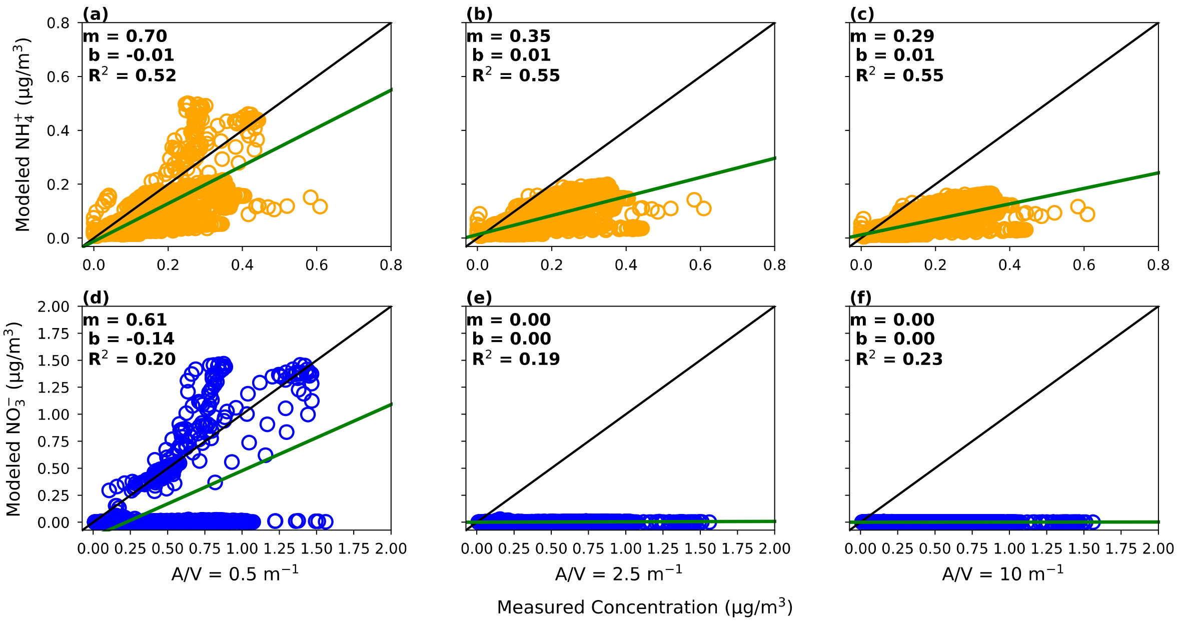

Figure S22. One-to-one plots comparing modeled NH_4^+ concentrations in the room to NH_4^+ concentration measurements are shown in the top row. One-to-one plots comparing modeled NO_3^- concentrations in the room to NO_3^- concentration measurements are shown in the bottom row. IMAGES was run with the measured temperature and RH conditions. The area-to-volume ratio in the room was set to either 0.5 m^{-1}(left column), 2.5 m^{-1}(middle column), or 10 m^{-1} (right column).

References

1. Claflin MS, Pagonis D, Finewax Z, et al. An in situ gas chromatograph with automatic detector switching between PTR- and EI-TOF-MS: isomer-resolved measurements of indoor air. *Atmospheric Measurement Techniques.* 2021;14(1):133-152.
2. Finewax Z, Pagonis D, Claflin MS, et al. Quantification and source characterization of volatile organic compounds from exercising and application of chlorine-based cleaning products in a university athletic center. *Indoor Air.* 2021;31(5):1323-1339.
3. Katz EF, Guo HY, Campuzano-Jost P, et al. Quantification of cooking organic aerosol in the indoor environment using aerodyne aerosol mass spectrometers. *Aerosol Science and Technology.* 2021;55(10):1099-1114.
4. Liu C, Wang H, Guo H. Redistribution of PM2.5-associated nitrate and ammonium during outdoor-to-indoor transport. *Indoor Air.* 2019;29(3):460-468.

5. Rickards AMJ, Miles REH, Davies JF, Marshall FH, Reid JP. Measurements of the Sensitivity of Aerosol Hygroscopicity and the κ Parameter to the O/C Ratio. *The Journal of Physical Chemistry A.* 2013;117(51):14120-14131.
6. Pye HOT, Nenes A, Alexander B, et al. The acidity of atmospheric particles and clouds. *Atmos Chem Phys.* 2019;20(8):4809-4888.
7. Drewnick F, Hings SS, Alfarra MR, Prevot ASH, Borrmann S. Aerosol quantification with the Aerodyne Aerosol Mass Spectrometer: detection limits and ionizer background effects. *Atmospheric measurement techniques.* 2009;2(1):33-46.
8. AG T. Specifications and Limits of Detection. 2023; https://www.tofwerk.com/products/clearfab-amc/specifications/.
9. INC P. PICARRO G2401 GAS CONCENTRATION ANALYZER. 2023; https://www.picarro.com/g2401_gas_concentration_analyzer.
